# Genomic selection for resistance to one pathogenic strain of *Vibrio splendidus* in blue mussel *Mytilus edulis*

**DOI:** 10.3389/fgene.2024.1487807

**Published:** 2025-01-03

**Authors:** Munusamy Ajithkumar, Jonathan D’Ambrosio, Marie-Agnès Travers, Romain Morvezen, Lionel Degremont

**Affiliations:** ^1^ Ifremer, Ressources Biologiques et Environnement (RBE)-ASIM, La Tremblade, France; ^2^ SYSAAF, Station LPGP/INRAE, Campus de Beaulieu, Rennes, France; ^3^ IHPE, CNRS, Ifremer, Université de Montpellier, University Perpignan Via Domitia, Montpellier, France

**Keywords:** mussels, mortality, breeding program, prediction accuracy, GWAS, linkage disequilibrium, *Vibrio*

## Abstract

**Introduction:**

The blue mussel is one of the major aquaculture species worldwide. In France, this species faces a significant threat from infectious disease outbreaks in both mussel farms and the natural environment over the past decade. Diseases caused by various pathogens, particularly *Vibrio* spp., have posed a significant challenge to the mussel industry. Genetic improvement of disease resistance can be an effective approach to overcoming this issue.

**Methods:**

In this work, we tested genomic selection in the blue mussel (*Mytilus edulis*) to understand the genetic basis of resistance to one pathogenic strain of *Vibrio splendidus* (strain 14/053 2T1) and to predict the accuracy of selection using both pedigree and genomic information. Additionally, we performed a genome-wide association study (GWAS) to identify putative QTLs underlying disease resistance. We conducted an experimental infection involving 2,280 mussels sampled from 24 half-sib families containing each two full-sib families which were injected with *V. splendidus*. Dead and survivor mussels were all sampled, and among them, 348 dead and 348 surviving mussels were genotyped using a recently published multi-species medium-density 60K SNP array.

**Results:**

From potentially 23.5K SNPs for *M. edulis* present on the array, we identified 3,406 high-quality SNPs, out of which 2,204 SNPs were successfully mapped onto the recently published reference genome. Heritability for resistance to V. splendidus was moderate ranging from 0.22 to 0.31 for a pedigree-based model and from 0.28 to 0.36 for a genomic-based model.

**Discussion:**

GWAS revealed the polygenic architecture of the resistance trait in the blue mussel. The genomic selection models studied showed overall better performance than the pedigree-based model in terms of accuracy of breeding values prediction. This work provides insights into the genetic basis of resistance to *V. splendidus* and exemplifies the potential of genomic selection in family-based breeding programs in *M. edulis*.

## 1 Introduction

Aquaculture is one of the fastest-growing food production industries, with an annual production of 130.9 million tons and a value of approximately 312.8 billion USD, while also maintaining a lower carbon footprint compared to terrestrial animals (Norman et al., 2019; FAO, 2024). Mussels are considered one of the major bivalve species cultured worldwide, with France ranking as the second largest European producer, producing 71,311 tons in 2022 (FAO, 2024). Two species as well as their hybrids are cultivated in France: the blue mussel *Mytilus edulis,* and the Mediterranean mussel *Mytilus galloprovincialis*. Production is distributed along the English Channel to the southwest coastline of France, and Mediterranean shores (Prou and Goulletquer, 2002). *Mytilus* species has been widely cultured due to its strong environmental adaptability, high nutritious value, and consumer preference (Prou and Goulletquer, 2002; Suplicy, 2020). French mussel production entirely relies on wild spat collection, mainly in Pays de Loire and in Nouvelle-Aquitaine regions (Prou and Goulletquer, 2002). Consequently, the French cultivated mussels are not genetically selected through selective breeding programs.

Massive mortality outbreaks have been reported in various bivalves species including mussels worldwide, significantly impacting production, causing economic losses, and negatively affecting ecosystems, with the cause often remaining unidentified (Degremont et al., 2007; Bódis et al., 2014; Burdon et al., 2014; Soon and Ransangan, 2019; Capelle et al., 2021; Lupo et al., 2021; Ericson et al., 2023). Since 2014, abnormal mussel mortality (AMM) outbreaks have been reported in French mussel farms, with mortality rates ranging from 10% to 99% depending on sites, seasons or years with peak mortality observed in the springs of 2014, 2016, and 2018 (Polsenaere et al., 2017; Degremont et al., 2019; Normand et al., 2022). Previous studies have identified several factors linked to AMM outbreaks, including seawater characteristics, pollution, mussel characteristics, culture practices, pathogens and climate change (review by Lupo et al., 2021). Various strains of *Vibrio splendidus* isolated from moribund mussels at sites affected by mass mortality have been linked to the AMM outbreaks (Bechemin et al., 2015; Ben Cheikh et al., 2016). *Vibrio splendidus* is a complex species comprising multiple strains, ranging from highly virulent to relatively innocuous (Ben Cheikh et al., 2016). Some virulent strains have been shown to be highly pathogenic to blue mussels, causing high mortality rates up to 90% within a week in experimental challenges (Ben Cheikh et al., 2016; Oden et al., 2016; Ben Cheikh et al., 2017). The virulence of these strains can vary based on several factors including mussel characteristics, environmental conditions, and seasons (Charles et al., 2020). While *V. splendidus* is not the direct cause of AMM, its consistent association with mortality outbreaks suggests it may play a contributory role under specific conditions. Recent studies on bivalve immune responses often lack a validated understanding of immune effectors or pathways, reflecting their reliance on innate rather than adaptive immunity, limiting the efficacy of vaccination strategies (Allam and Raftos, 2015; Rey-Campos et al., 2019). Selective breeding could be a useful approach to enhance the innate immune responses in bivalves (Degremont et al., 2015; Hollenbeck and Johnston, 2018). Understanding genetic basis of disease resistance is critical for its improvement through selective breeding.

Over the past decades, many commercially important bivalve species have demonstrated significant genetic improvement through mass selection, due to their high fecundity and short generation intervals (Gjedrem and Rye, 2018; Tan et al., 2020). Mass selection has been carried for growth traits in Chilean blue mussel *Mytilus chilensis* (Toro et al., 2004a; Toro et al., 2004b), and ploidy status for Mediterranean mussel *M. galloprovincialis* (Ajithkumar et al., 2025). Lately, a mass selection scheme implemented for resistance to AMM outbreaks in the blue mussel *M. edulis* resulted in a 34%–48% increase in survival after one generation of selection (Degremont et al., 2019). Although mass selection is effective, it may quickly lead to inbreeding if genetic diversity is not properly monitored (Hu et al., 2022). However, family based selective breeding programs have been initiated as an alternative strategy to mass selection, to estimate breeding values by combining phenotypic information and pedigree. These programs have targeted various traits across different mussel species, such as growth in *M. edulis* (Mallet et al., 1986), *M. galloprovincialis* (Nguyen et al., 2014; Pino-Querido et al., 2015; Díaz-Puente et al., 2020), *M. chilensis* (Alcapán et al., 2007; Guiñez et al., 2017), *Hyriopsis cumingii* (Jin et al., 2012; Bai et al., 2017), *Perna calaniculus* (Camara and Symonds, 2014); shell nacre color in *H. cumingii* (Bai et al., 2017); toxin accumulation and mantle color in *M. galloprovincialis* (Pino-Querido et al., 2015); and survival in *M. edulis* (Mallet et al., 1986).

Accurate estimations of breeding values are crucial for developing a successful breeding program and predicting the responses of traits of interest to selection. Advances in high-throughput genotyping technology have now made it possible to implement genomic selection effectively in aquaculture species (Boudry et al., 2021). Genomic selection is especially beneficial for traits that are costly or difficult to measure, such as flesh quality or disease resistance, and it can achieve similar or higher accuracies in estimated breeding values (EBVs) with less phenotypic data compared to traditional family-based selection (Regan et al., 2021; Yáñez et al., 2023). Furthermore, genomic selection enhances genetic gain by capturing genetic variation both within and between families (Boudry et al., 2021). Next-generation sequencing and genotyping-by-sequencing tools have been developed to detect genome-wide molecular markers for oyster, clam, abalone and scallop (Jiao et al., 2014; Ren et al., 2016; Wang et al., 2016; Nie et al., 2017; McCarty et al., 2022; Yang et al., 2022). However, these methods may not provide reliable markers for testing across different populations (e.g., between the training and breeding populations) because they sequence a large number of randomly distributed regions, and their effectiveness can vary with DNA quality, limiting their ability to produce consistent genomic analyses (Davey et al., 2011; Boudry et al., 2021). Alternatively, SNP arrays have been developed for several commercially important bivalve species, including silver-lipped pearl oyster (*Pinctada maxima)*, with an Illumina ∼3k iSelect custom array (Jones et al., 2013a), the medium density bi-species (Pacific oyster *Crassostrea gigas* and European flat oyster *Ostrea edulis*) 57K SNP array (Gutierrez et al., 2017), the Pacific oyster (*C. gigas)*, with a 190K SNP array (Qi et al., 2017), the Eastern Oyster (*C. virginica*), with a high density 566K and 66K SNP array (Guo et al., 2023), or medium density multi-species (*M. edulis*, *M. galloprovincialis*, *M. trossulus*, and *M. chilensis*) 60K SNP array (Nascimento-Schulze et al., 2023), all of which rely on commercial chip products with fixed sets of markers and specific genotyping platforms. SNP arrays have been applied to a range of aquaculture species for various purposes, such as uncovering the genetic architecture of traits, implementing genomic selection, characterizing genetic resources, pedigree monitoring, sex-determination, and inbreeding management. However, their use in bivalves has been relatively limited (Gutierrez et al., 2020; Jourdan et al., 2023).

In aquaculture, the salmon industry has been leading the way in genomic selection for several years (Odegård et al., 2014; Tsai et al., 2015; Correa et al., 2017; Robledo et al., 2018; Ajasa et al., 2024). To date, more frequently aquaculture species are following this trend such as rainbow trout *Oncorhynchus mykiss*, European sea bass *Dicentrarchus labrax*, Gilthead Sea Bream *Sparus aurata*, Nile tilapia *Oreochromis niloticus*, Channel catfish *Ictalurus punctatus* or whiteleg shrimp *Litopenaeus vannamei* [see for review (Houston et al., 2020; Boudry et al., 2021; Song et al., 2022; Yáñez et al., 2023)]. The recent advancements in genotyping tools have paved the way for exploring the potential of genomic selection in bivalves. Genomic selection has been investigated in the Portuguese oyster for morphometric traits, edibility traits and disease traits (Vu et al., 2021), in the American oyster for low salinity tolerance (McCarty et al., 2022),in the silver-lipped oyster for pearl quality traits (Zenger et al., 2019) and growth traits has been studied in the triangle sail mussel (Wang et al., 2022), European flat oyster (Penaloza et al., 2022) and Pacific oyster (Gutierrez et al., 2018; Jourdan et al., 2023). A few studies have been conducted in oysters showing the increase in accuracy of genomic selection over pedigree-based approaches for difficult to measure traits, such as disease resistance, meat content and color traits (Gutierrez et al., 2018; Gutierrez et al., 2020; Jourdan et al., 2023). To date, trials with low-density panels to reduce genomic evaluation costs have been conducted in several aquaculture species, indicating that developing cost-effective strategies for genomic selection will be pivotal in shaping modern aquaculture breeding programs (Kriaridou et al., 2020; Penaloza et al., 2022).

The primary aim of our study was to evaluate the potential of genomic selection for resistance to one pathogenic strain of *V. splendidus* in *M. edulis*. Using a multi-species Axiom Affymetrix 60K SNP array (Nascimento-Schulze et al., 2023), we first characterized the genetic structure and linkage disequilibrium of the blue mussel population. We then estimated genetic parameters for resistance to *V. splendidus* and performed GWAS to investigate its genetic architecture. Finally, we compared genomic and pedigree-based selection accuracies to optimize breeding programs.

## 2 Materials and methods

### 2.1 Family production

The 48 families of *M. edulis* used in this study are detailly described in (Ajithkumar et al., 2024b). Briefly, three wild mussel populations were sampled, two from the Atlantic coast (OLE-PON and YEU_001), and a third from the North of France (WIM), and transferred to the Ifremer hatchery in La Tremblade in the fall of 2016. Each mussel population was cleaned and placed in separate tanks containing unheated UV-treated, and filtered seawater (400 L per hour). To favor gametogenesis, mussels were fed a cultured phytoplankton diet (*Isochrysis galbana*, *Tetraselmis suecica*, and *Skeletonema costatum*). Two sets of crosses were performed in January 2017 (set 1) and in February 2017 (set 2). For each population, 100 mussels were individually placed in 400 mL beakers, and spawning was triggered by alternating cold (10°C) and warm seawater (20°C). Depending on the ripeness of the mussels and the sex ratio, four males for OLE-PON, and 11 males for YEU_001 were used in set 1, while 9 males were used for WIM in set 2. Within population, each male was mated with two females, producing in total 24 half-sib families, each containing two full-sib families. These 48 full-sib families consist of 8, 22 and 18 families from OLE-PON, YEU_001 and WIM population, respectively, all should be considered as outbred. Each full-sib family was grown separately in 30 L tanks filled with filtered and UV-treated seawater at 20°C until the pediveliger stage. Then, downwelling system were used until mussels reached 1 cm. At that size, they were transferred to our nursery in Bouin in April and May 2017 for set 1 and set 2, respectively. For each family, 1,000 spat were maintained in 15 L SEAPA^©^ baskets, and all families were raised in a 20 m³ concrete raceway until the start of the experiment, which occurred in July 2018. More detailed on the larval and nursery culture are provided in Ajithkumar et al. (2024b).

### 2.2 Experimental infection and phenotyping

Detailed step-by-step protocol of the experimental infection is given in Ajithkumar et al. (2024b). Briefly, two experimental infections (EI_1 and EI_2) were conducted in July 2018. Among the 48 families (mean individual total weight of approximately 5 g), 24 families were randomly sampled and tested in El_1, and the others were tested in El_2. Additionally, a third experimental infection (EI_3) was performed, involving the same 24 families from El_1, to increase the genotype sample size due to some samples from El_1 were lost. Finally, three families tested in El_2 were also tested in El_1, leading to a total of 27 families tested in El_1, and one of those three was also tested in El_3 indicating that only one family was tested in each of the three experimental infections (see Supplementary Table S1 for details). For each experimental infection and family, a highly pathogenic strain of *V. splendidus* (strain 14/053 2T1) isolated during AMM outbreak in 2014 was injected in 30 mussels to investigate their resistance to this pathogen. This particular strain was selected for its high virulence, the highest among the 50 strains tested, and it belongs to a specific pathogenic group. The lethal dose-50 (LD_50_) was determined prior to the experimental infection by injecting varying concentrations of *Vibrio splendidus* strain into a large group of mussels. The selected dose was close to the LD_50_, ensuring adequate variance in the response for effective assessment of resistance. First, mussels were anesthetized using MgCl_2_ (50 g per L), and 50 μL of bacterial solution (10^9^ bacteria/mL) was injected into the muscle. Then, ten injected mussels per family, for all the 24 families of one set were hold in one 120 L tank containing UV-filtered seawater. Three replicate tanks were used and, in each tank, water recirculation was maintained using a TECO®pump (Ravenna, Italy), which also maintained the seawater temperature at 17°C. Dead mussels were counted and sampled daily up to 72 h post-injection. The adductor muscle/gills of the dead mussels during the experiment and the surviving mussels at the end of the experiment were collected using scalpels disinfected with 70% ethanol and stored in 1.5 mL sterile tubes at room temperature.

### 2.3 Genotyping and quality control

Among the dead and alive animal sampled, around 14–15 mussels were genotyped per family (ranging from 13 to 16) (Table 1; Supplementary Table S1). The number of dead and alive per family was calculated in proportion to the mortality observed within each family in El_1 and in El_2. As the number of animals sampled in El_1 was not sufficient, 158 dead mussels and 35 live mussels were sampled from EI_3 (Supplementary Table S1). In total, 768 individuals were genotyped; 348 dead, 348 alive, and the remaining 72 were their parents (48 dams and 24 sires). DNA extraction and genotyping were performed by the Gentyane INRAE Platform (Clermont-Ferrand, France) using the multi species medium-density 60K SNP-array, Axiom_Myt_v1_r1 (Thermo Fisher Scientific, Waltham, Massachusetts, United States), which comprises 23,252 markers for *M. edulis* (Nascimento-Schulze et al., 2023). Quality controls on the 60K SNPs from the SNP array and genotyped individuals were performed as described in D'Ambrosio et al. (2019). Firstly, genotypes of all individuals were analyzed using the Axiom Analysis Suite software (AxAS; v.4.0.3.3) with the default best practice workflow suggested by the manufacturer, with few threshold modifications, which includes individual quality control and SNP quality control analysis (Dish Quality Control ≥0.20; Quality Control call rate ≥90; percent of passing samples ≥98; average call rate for passing samples ≥92%; call rate cutoff ≥95; Fisher’s Linear Discriminant ≥2.6). Consequently, 7,476 polymorphic SNPs were retained for further analysis. Subsequently, final quality control was performed using PLINK v1.9 software (Chang et al., 2015). Two individuals with an identity-by-descent value over 0.90 were considered as duplicated and both individuals were removed from the analysis. Only SNPs with a minor allele frequency (MAF) higher than 0.01 and those passing the Hardy-Weinberg equilibrium test (p-value <0.0000001) in the genotyped mussels were retained. After the quality control, data comprised of a total of 766 genotyped individuals for 3,406 SNPs.

**TABLE 1:** Summary of the experimental infection using the pathogenic strain 14/053 2T1 of *Vibrio splendidus* in *Mytilus edulis*.

	Phenotyped	Genotyped
Number of families	48	48
Total number of mussels	2,280	768
Mean number of mussels/family	47.5	14.5 (Min:13; Max:16)
Mean mortality	47.3%	50%

### 2.4 Parentage assignment

Parentage assignment was performed in the R package APIS (Griot et al., 2020) with a mismatch number set to 5%. The best 1,471 SNPs, selected with call rate greater than 90% and MAF value greater than 0.1 were used. Parentage assignment allowed the reconstruction of the pedigree of 647 offspring with assignment rates reaching 93.2% of the mussels having both parents assigned, while the remaining 47 mussels potentially from outside the cross-mating design, which were excluded from following analyses.

### 2.5 Genetic structure of the population

To evaluate potential genetic sub-structuring of populations and any associated biases, a principal component analysis (PCA) was performed using PLINK 1.9 (Chang et al., 2015) and the genetic structure was visualized using in RStudio (Team, 2024). Three individuals were identified as outliers beyond the population structure and were subsequently excluded from further analysis. Genetic differentiation between populations was measured through pairwise fixation index (F_ST_) estimates using PLINK 1.9 (Chang et al., 2015).

### 2.6 SNP mapping, genome coverage and linkage disequilibrium estimation

All markers of the array along with their flanking regions were blasted using a BLASTn^®^ procedure on the reference genome (*Mytilus edulis* genome assembly, xbMytEdul2, GenBank accession number: GCA_963676595.2). To map SNPs, considering the high polymorphism in the mussel genome, four mismatches were allowed over a length of around 71 base pairs. Only SNPs mapping to a unique position on the reference genome were retained for the subsequent stage of quality control as mentioned in previous section. Out of the 3,406 SNPs, only 2,204 matched our mapping criteria and were successfully positioned on the reference genome (Supplementary Table S2).

The pairwise linkage disequilibrium (LD) analysis was performed between all SNPs and adjacent markers for each linkage group and population to determine LD decay within the genome of *M. edulis* using Plink 1.9 (Chang et al., 2015).

### 2.7 Estimation of genetic parameters

#### 2.7.1 PBLUP

Estimated breeding values, variance components, and heritability were calculated using the BLUPF90 software package (Misztal et al., 2014) through two different approaches: a linear mixed model with AIREMLF90 (Misztal et al., 2014) for assessing the trait on the observed scale, and a Gibbs analyses with THRGIBBS1F90 (Tsuruta and Misztal, 2006) for evaluating it on the underlying scale, based on pedigree-based relationship.

Y=Xβ+Zμ+e

where Y is the binary mortality outcome at the end of the experiment (1 = dead, 2 = alive) of mussel, β is the vector of fixed effects, including set of crosses (set 1, set 2), population origins (OLE-PON, WIM, YEU_001), and replication of the experimental infection (EI_1, EI_2 and EI_3). $\mu_{i}$ is the vector of additive genetic effect of the animal, following a normal distribution μ ∼ N $\left(0,A\sigma_{a}^{2}\right)$ , where A is the pedigree relationship matrix, and $\sigma_{a}^{2}$ is the additive genetic variance. e is the vector of random residuals, assumed to be distributed as $\left.e∼N(0,I\sigma_{e}^{2}\right)$ where I is an identity matrix and $\sigma_{e}^{2}$ is the residual variance. X and Z are known incidence matrices relating observations to the fixed and random effects mentioned above. Random tank effect was removed from the model due to the lack of significance.

The EBV were estimated using BLUPF90 package and the variance components using AIREMLF90 and THRGIBBS1F90 programs. With the threshold model, the variance components were estimated using a Gibbs sampler with 100,000 iterations, 10,000 of burn-in and one sample was kept every 10 iterations for posterior analysis. Variance components were estimated using the average information restricted maximum likelihood algorithm (Gilmour et al., 1995). The h^2^ for linear model on observed scale transferred into underlying scale using the formulae from Dempster and Lerner (1950).

Heritability ( $h^{2}$ ) was estimated as: $h^{2}=\frac{\sigma_{a}^{2}}{\sigma_{a}^{2}+\sigma_{e}^{2}}$

#### 2.7.2 GBLUP

Estimated heritability was calculated using animal linear model with AIREMLF90 and animal threshold model with Gibbs sampling using THRGIBBS1F90. The GBLUP model uses the same approach as the PBLUP model, but A replaced by G . Here, G is the genomic relationship matrix. The matrix G was computed as described by VanRaden (2008).

$$G=\frac{ZZ^{\prime }}{\sum_{i}^{m}2p_{i}\left(1-p_{i}\right)}$$

where Z is a matrix of centered genotypes $\left(0-2p\right.$ = homozygous, 1−2p= heterozygous, 2−2p= homozygous), $p_{i}$ is the frequency of the reference allele for the $i^{th}$ marker, and m is the total number of markers.

The population-specific analysis was performed using SNP markers to estimate heritability within population, and genetic correlation between populations. A high genetic correlation (0.99) was observed between populations (Supplementary Table S3).

#### 2.7.3 ssGBLUP

Estimated heritability was calculated using animal linear model with AIREMLF90 and animal threshold model with Gibbs sampling using THRGIBBS1F90. The single-step GBLUP (ssGBLUP) model enhances the PBLUP and GBLUP model by fitting the H matrix, which integrates both genomic and pedigree data (Aguilar et al., 2010). The inverse of the H matrix was constructed as follows:

$$H^{-1}=A^{-1}+\left[\begin{array}{cc} 0 & 0\\ 0 & {\left(0.95G+0.05A_{22}\right)}^{-1}-A_{22}^{-1} \end{array}\right]$$

where G is as described above and A_22_ is the pedigree-based relationship matrix for genotyped animals.

### 2.8 Genome-wide association study 

To identify SNPs associated with resistance to *V. splendidus*, a genome wide association study (GWAS) was performed using a mixed linear model association through ssGBLUP analysis. The postGSF90 module (Misztal et al., 2014) from the BLUPF90 package was used to estimate the effects of the SNPs ( ${\hat{a}}_{i}$ ) based on the genomic breeding values ${\hat{g}}_{i}$ predicted for the genotyped animals. The SNP effects were estimated according to the following equation:

$${\hat{a}}_{i}=dZ^{\prime }{\left[ZdZ^{\prime }\right]}^{-1}{\hat{g}}_{i}$$

where d is the vector of weights associated with the SNP effects and Z is the incidence matrix relating SNP effects to genomic breeding values.

Estimates of SNP effects ( ${\hat{a}}_{i}$ ) can be used to estimate the proportion of additive genetic variance of each SNP effect:

$${\%V}_{a}=\frac{2p\left(1-p\right)\;{{\hat{a}}_{i}}^{2}}{\sigma_{a}^{2}}*100$$

with $\sigma_{a}^{2}$ the total genetic variance estimated using the linear mixed model with postGSF90 and p the minor allele frequency of the target SNP.

### 2.9 Prediction accuracy

Prediction accuracy for the PBLUP, GBLUP, and ssGBLUP models was assessed using the ‘leave-one-out’ method, implemented with a linear model in BLUPF90 (Resende et al., 2012; Kristensen et al., 2018). In this approach, each observation is systematically excluded one at a time. The model is then trained on the remaining data, and the (G)EBV for the excluded individual is predicted by masking its phenotype.

The accuracy (r) of prediction was computed as the correlation between the (G) EBVs and the corrected phenotype ( $\hat{y}$ ) of the mussel divided by the square root of the heritability, using the formula:

$$r=\frac{\left[\left(G\right)\text{EBV},\hat{y}\right]}{\sqrt{h^{2}}}$$

The heritability value $\left(h^{2}\right)$ used in this analysis was calculated using the variance components ( $\sigma_{a}^{2}$ and $\left.\sigma_{e}^{2}\right)$ from the ssGBLUP model.

#### 2.9.1 Evaluation of the effect of SNP density on genomic predictions (GP)

SNP panels of varying densities were assessed by selecting subsets from the full QC-filtered SNP panel for each dataset. Panels of the following densities were tested: 500 SNPs, 1,000 SNPs, 1,500 SNPs, annotated SNPs (2,204), and all high-quality SNPs (3,406). SNPs for each panel were randomly sampled within each chromosome, with the number of SNPs chosen from each chromosome being proportional to the total number of high-quality SNPs per chromosome. To mitigate biases, we randomly generated five different SNP panels for each SNP density.

## 3 Results

### 3.1 *Vibrio* challenge

**FIGURE 1:**
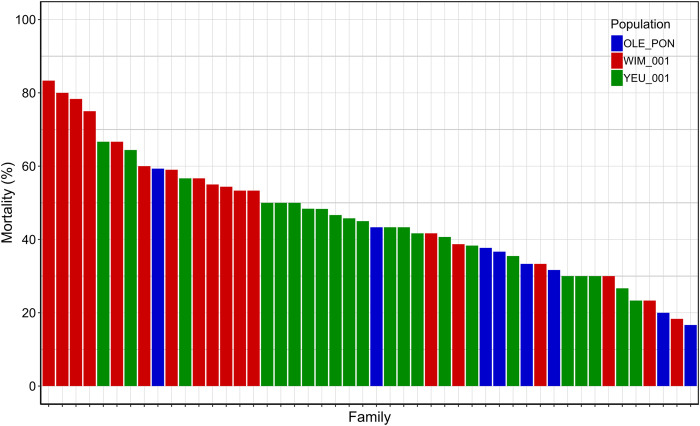
Final cumulative mortality 72 h post-injection for each family. Each bar represents a family and each color represent a population.

The cumulative mortality rate 72 h post-injection was 47%. At endpoint, mortality rates were 63% for EI_1, 41% for EI_2, and 37% for EI_3. Among mussel populations, the WIM population (54%) showed higher susceptibility to *V. splendidus* compared to the YEU_001 (45%) and OLE-PON (37%) populations (Supplementary Table S4). Mortality rates varied significantly among families upon exposure to *V. splendidus*, ranging from 17% to 83%. The mean mortality rates for all families are depicted in Figure 1.

### 3.2 Population structure

**FIGURE 2:**
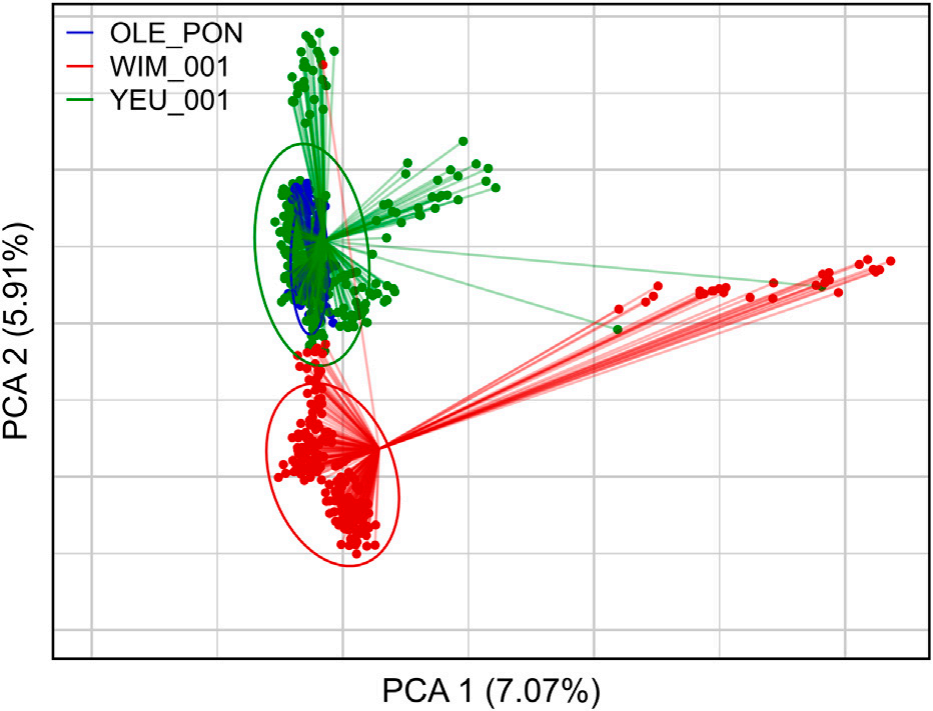
First two axes and associated variances of the principal component analysis (PCA) of the genetic diversity among the three populations of *Mytilus edulis*. The ellipses are constructed with axes defined as 1.5 times the standard deviation of the projections of individual coordinates on the axes. PCA was performed with 647 individuals and 3,406 SNPs.

**FIGURE 3:**
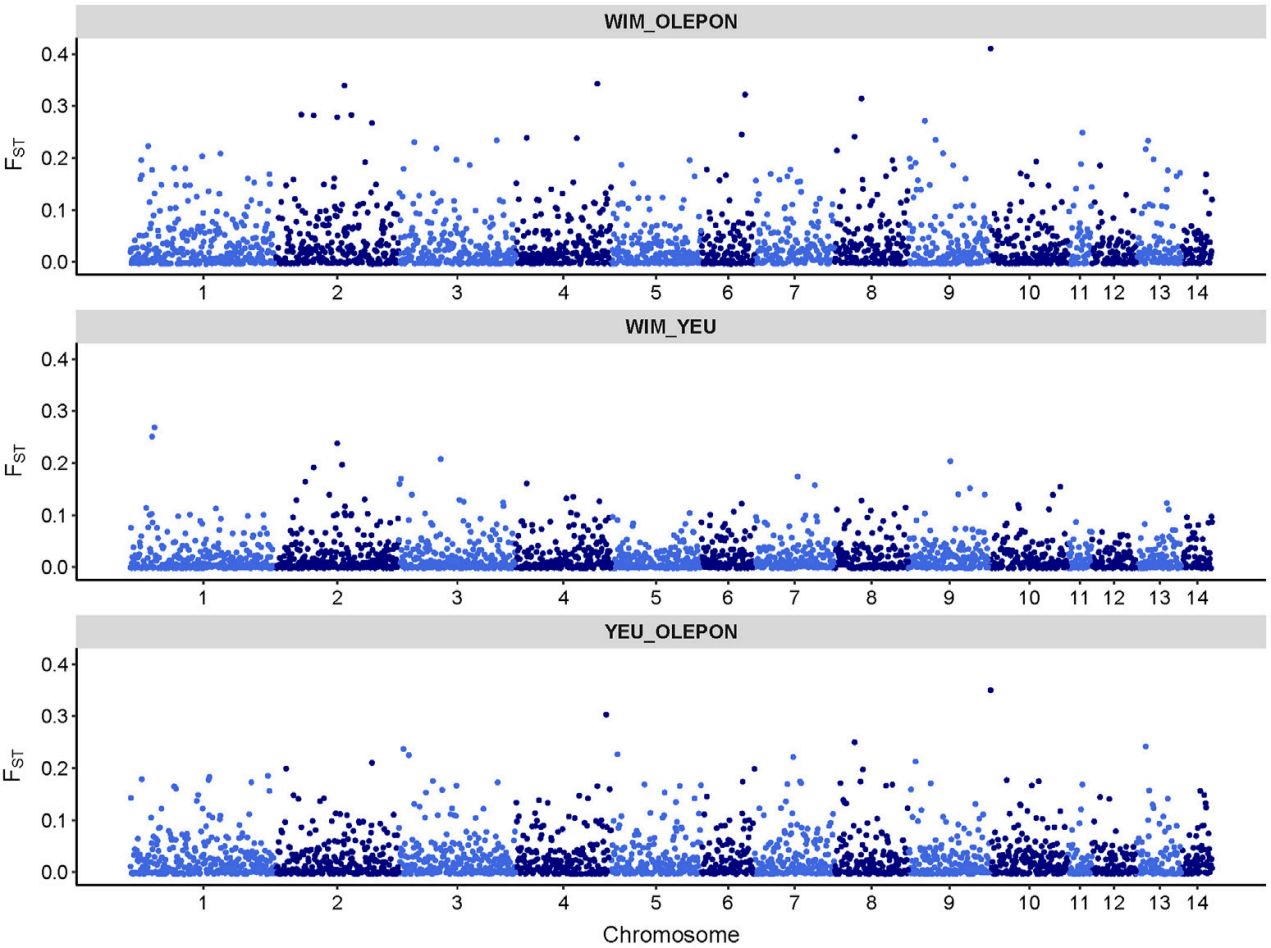
Genomic distribution of fixation index (F_ST_) values as a function of chromosome position in the mussel genome for different studied population.

Figure 2 illustrates the results of the principal component analysis (PCA), revealing the population structure of the mussel population. The first two PCA axes collectively account for over 15% of the total genetic variation. The YEU_001 and OLE-PON populations were generally homogeneous, although there were few stratifications within YEU_001. The WIM population have quite different genetic background from others, with two families whose offspring showed even greater isolation. Nevertheless, F_ST_ analysis revealed low genetic differentiation between populations. The mean genetic distances between populations are shown in Table 2, with F_ST_ values ranging from 0.02 to 0.03, suggesting genetic similarity across all three populations (Figure 3).

**TABLE 2:** Pairwise F_ST_ between populations of *Mytilus edulis*.

	WIM	YEU_001
OLE-PON	0.03	0.03
WIM		0.02

### 3.3 SNP mapping and genome coverage

2,204 SNPs were positioned on the reference genome, resulting a loss of 1,202 SNPs. The positions of markers on the chromosomes are illustrated in Supplementary Figure S1. The average SNP density per megabase (Mb) ranges from 0.57 to 2.37, varying among chromosomes and within chromosome (Supplementary Table S5). Approximately, only 9% of all 1 Mb segments contain more than 5 SNPs. SNP density exhibits non-uniformity throughout the genome, with each chromosome demonstrating varying densities. The lower marker density results in greater mean average distances between adjacent SNPs, ranged from 421 kb to 1739 kb depending on the chromosome.

### 3.4 Linkage disequilibrium analysis

**FIGURE 4:**
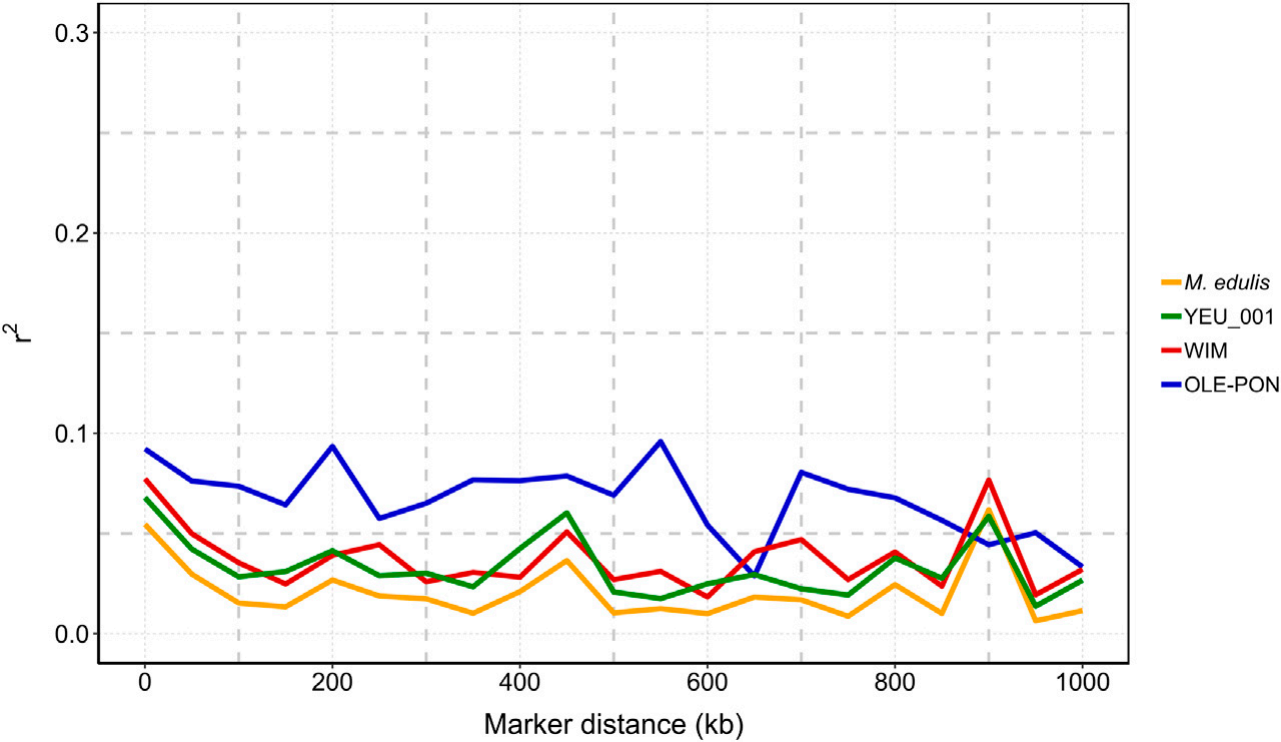
Linkage disequilibrium (*r* ^2^) decay with physical distance between markers in each population and overall challenged to *Vibrio splendidus*. The *X*-axis is the physical location, and the *Y*-axis is the linkage disequilibrium value (*r* ^2^).

Figure 4 illustrates that linkage disequilibrium (LD) decreases sharply as the distance between pairs of SNPs increases, with the most rapid decline occurring within the first 100 kb. Beyond this range, LD continues to decline and becomes more variable. The OLE-PON population consistently shows higher LD throughout the genome compared to other populations. On average, the LD values (*r* ^2^) for SNPs less than 15 kb apart are 0.12 for OLE-PON, 0.10 for WIM, and 0.06 for YEU. Linkage disequilibrium values are generally low between adjacent SNPs for all the chromosomes, where distances between adjacent SNPs are larger.

### 3.5 Heritability

The estimates of heritability using the linear and Gibbs sampling models are summarized in Table 3. Pedigree-based heritability estimates for resistance to *V. splendidus* in *M. edulis* ranged from 0.22 to 0.31. Genomic heritability was slightly higher, varying between 0.33 and 0.36. The ssGBLUP based estimated heritability ranging from 0.28 to 0.33, which combines genomic and pedigree information.

**TABLE 3:** Heritability for resistance to *Vibrio splendidus* in *Mytilus edulis*.

Method	Model	Relationship matrix	h^2^ (±se)
PBLUP	Linear	A	0.22 (0.06)
	Gibbs sampling	A	0.31 (0.05)
GBLUP	Linear	G	0.33 (0.11)
	Gibbs sampling	G	0.36 (0.05)
ssGBLUP	Linear	H	0.28 (0.08)
	Gibbs sampling	H	0.33 (0.05)

A, pedigree based relationship matrix; G, genomic based relationship matrix; H, genomic and pedigree combined relationship matrix; Linear, Linear mixed model; h^2^, heritability. The h^2^ for linear model on observed scale transferred into underlying scale using the formulae from Dempster and linear (1950).

### 3.6 Genetic architecture

**FIGURE 5:**
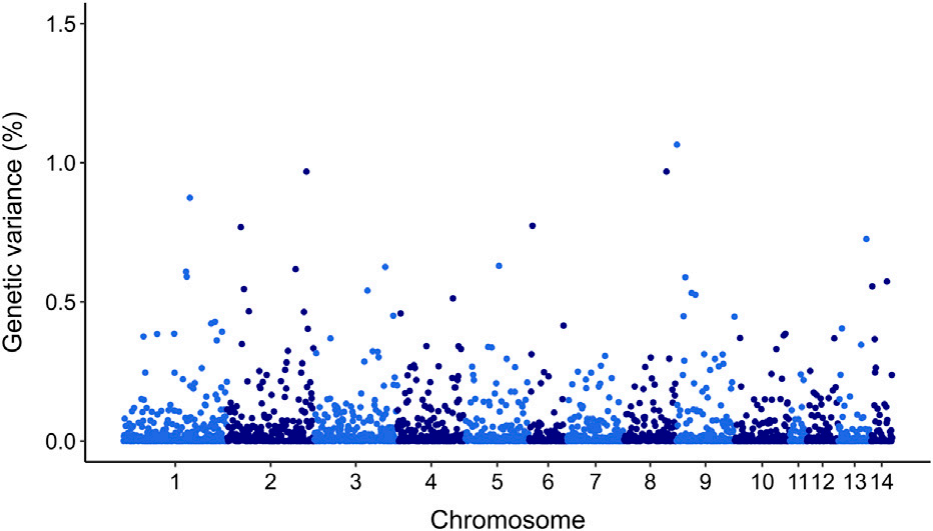
Manhattan plot of genetic variance explained by each SNP for resistance to *Vibrio splendidus* in *Mytilus edulis* using ssGBLUP approach. In *X*-axis SNP per chromosome and *Y*-axis percentage of genetic variance explained per each SNP.

GWAS for resistance to *V. splendidus* suggest that this trait is likely to be impacted by multiple genomic regions. However, there were 20 SNPs that explained >0.5% of additive genetic variance on chr 1, chr 2, chr 3, chr 4, chr 5, chr 6, chr 8, chr 9, chr 13, and chr 14 (Figure 5). However, none of these markers explained more than 1.06% of the additive genetic variance (Figure 5; Table 4).

**TABLE 4:** Summary of top five SNPs associated with resistance to *Vibrio splendidus* in GWAS analysis (ssGBLUP) ranked with respect to genetic variance. Position = Physical position of SNP on the chromosome; A1 and A2 = Minor and major alleles, respectively; MAF = Minor allele frequency; var A = percentage of additive genetic variance explained by SNP.

SNP_ID	Chromosome	Position	A1	A2	MAF	Var a (%)
AX-604710899	9	356,838	A	T	0.32	1.06
AX-604117866	8	67,449,025	T	C	0.35	0.97
AX-604122940	2	97,860,291	T	G	0.29	0.97
AX-604245954	1	94,565,421	A	C/T	0.43	0.87
AX-603828141	6	11,814,154	C	T	0.33	0.77

### 3.7 Prediction accuracy

**FIGURE 6:**
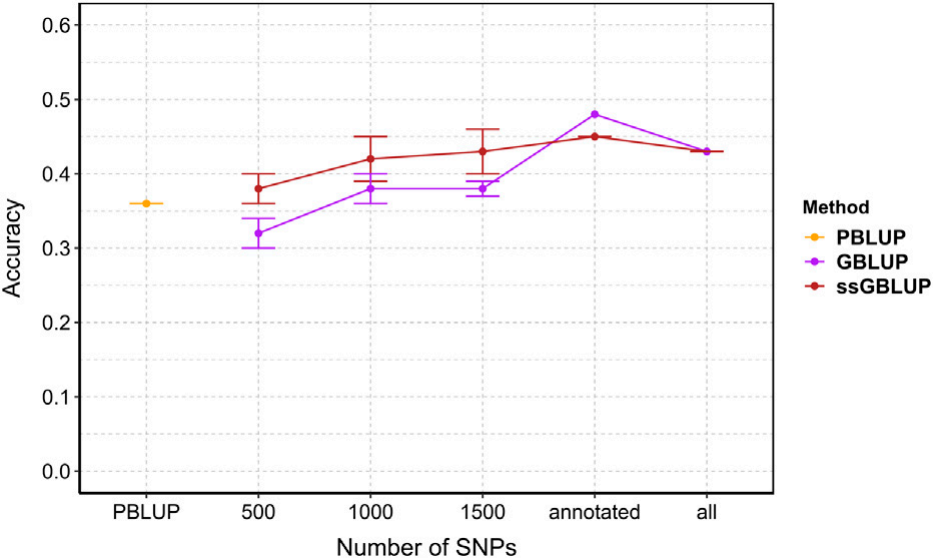
The estimated prediction accuracy of *Vibrio splendidus* resistance *in Mytilus edulis* using PBLUP, GBLUP and ssGBLUP across different marker densities. Each point is the average of 5 replicates. Error bars represent the standard error of the mean of 5 replicates. PBLUP - Pedigree based breeding values using all phenotyped animals, respectively. GBLUP - Genomic breeding values from only genotype animals, and ssGBLUP - Genomic breeding values from all genotyped and phenotyped animals obtained with a combined relationship matrix (H). Annotated: SNPs mapped onto the recently published reference genome (2,204); all: retained SNPs after quality control (3,406).

Accuracy with all data are 0.36, 0.43, 0.43 for PBLUP, GBLUP and ssGBLUP, respectively. Genomic selection (GBLUP and ssGBLUP) is better than PBLUP by 19%. Overall, prediction accuracy for genomic selection increased with the density of markers (Figure 6). Incorporating genomic information generally enhanced accuracy compared to pedigree-based estimation, except with 500 SNPs where PBLUP exhibited higher accuracy than GBLUP (Figure 6). With maximum training population and SNP subsets, genomic evaluation improved accuracy by 17%, 19%, 25%, and 19% for 1,000, 1,500, annotated (2,204), and all SNPs (3,406), respectively, compared to PBLUP. When comparing GBLUP and ssGBLUP models, the prediction accuracy was consistently favored the ssGBLUP model, except when using annotated SNPs in the GBLUP model (Figure 6).

## 4 Discussion

In our study, we aimed to demonstrate the feasibility of genomic selection in a mussel breeding program in France. We used a recently developed multi species medium-density 60K SNP-array (Nascimento-Schulze et al., 2023) to perform genomic analysis. Combining multiple populations is a common approach for multi-population GP and combined GWAS analysis (VanRaden et al., 2009; Lund et al., 2011; Begum et al., 2012). However, there are three criteria for using this approach: first, the trait of interest is measured in the same way in different populations and genotyping is done using same SNP array; second, there is no G × E interaction in different populations; and finally, the genetic distance between the populations is minimal (Lund et al., 2011; Begum et al., 2012; Gebreyesus et al., 2019). As our study meets these requirements, combined population analyses were performed.

### 4.1 Genotyping quality and genome covering by selected SNPs

To the best of our knowledge, our study is the first to use the multi species medium-density 60K SNP-array (Nascimento-Schulze et al., 2023) to estimate genetic and genomic parameters in blue mussel (*M. edulis*). After all quality controls, we identified high-quality SNPs from 23,252 SNPs present on the array across 768 individuals. The necessity for stringent filtering of genotyping data is highlighted by the prevalence of poor-quality markers. One of the distinguishing features of the *Mytilus* species complex is its high degree of heterozygosity and abundance of mobile elements, which pose challenges in accurately identifying polymorphic SNPs (Gerdol et al., 2020; Sun et al., 2017). Compounding this difficulty, the current SNP array was developed using a whole-genome low coverage approach, which can lead to loss of polymorphic sites, especially across genetically diverse populations. A prior study validating this SNP array found 89.8% of the 23,252 SNPs to be polymorphic due to the close genetic match between the populations used for development and validation (Nascimento-Schulze et al., 2023). In contrast, after quality control in our study, only 14.6% of the SNPs were retained as polymorphic, likely due to genetic differences between our sampled population and the one used for array creation. This discrepancy highlights the influence of population structure on SNP retention and substantial genetic complexity and high polymorphism exhibited in mussel genome (Gerdol et al., 2020; Smietanka et al., 2014). This complexity stems from its evolutionary history of extensive gene flow among related species and a genome characterized by significant dispensable content. Indeed, about 30% of genes in *Mytilus* are dispensable, likely impacting SNP distribution and usability across populations (Gerdol et al., 2020). Out of 23,252 SNPs identified in *M. edulis*, without any other quality control, only 16,213 (70%) were annotated on the recently published reference genome of *M. edulis*. Assembly errors in the reference genome may rise from several factors, such as exceptionally high genetic polymorphism levels, non-Mendelian segregation of marker loci in paired crosses, and a significant occurrence of null alleles in genetic markers (Hedgecock et al., 2015). If we consider the other quality controls done, only 2,204 SNPs (64.8% of polymorphic, good quality SNPs, 9.4% of the total available SNPs) mapping successfully. This limited SNP coverage and sparse distribution across the linkage map could potentially lead to the omission of QTLs in specific regions, particularly in regions where markers are underrepresented. To improve SNP array quality and increase the number of informative SNPs, future work could include strategies such as pooled sequencing with a greater number of individuals, incorporating genetically diverse populations, or performing high-coverage whole-genome resequencing numbers of individuals from each population (Nascimento-Schulze et al., 2023). These adjustments would increase the SNP array’s robustness, thereby improving genome coverage, QTL detection power, and marker-trait association accuracy in *M*. *edulis*.

The bi-species Axiom Affymetrix 57K SNP array has been used in Pacific oysters, where applying the AxAS software’s best practice workflow led to a notable reduction in the number of informative SNPs. Specifically, Gutierrez et al. (2018) reported 23,000 informative SNPs from 820 individuals, Vendrami et al. (2019) identified 21,499 SNPs from 232 individuals, and Jourdan et al. (2023) obtained 14,500 SNPs from 2,420 individuals. This reduction is largely attributed to the complex genetic structure of molluscs, stemming from the highly polymorphic nature of their genomes (Gerdol et al., 2020; Jiao et al., 2021; Song et al., 2021), and is further influenced by the genetic relationship between the training population used for array design and the breeding candidates in selective breeding program (Houston et al., 2020). However, recent studies on bivalves have demonstrated that a moderate number of high-quality markers (1,000–3,000) could suffice for accurate predictions (Gutierrez et al., 2018; Kriaridou et al., 2020; Penaloza et al., 2022).

### 4.2 Linkage disequilibrium

Linkage disequilibrium (LD) at the genome level plays a crucial role in the efficacy of breeding programs, influencing genetic variance and the accuracy of association analyses (Goddard and Hayes, 2009; Siol et al., 2017). In our study, values for *r* ^2^ ranged between 0.07 and 0.09 for SNPs within a distance of 10 kb and from 0.03 to 0.08 within 50 kb in the populations studied, suggesting limited short-range LD at current SNP marker densities. LD levels decreased to less than 0.05 at 100 kb in two populations. The high LD in OLE-PON is probably an overestimate due to a smaller effective population size (N_e_) than in the other populations. Overall, LD between adjacent markers within each population was predominantly less than 0.1 within 2 kb, indicating a rapid decline in LD within the blue mussel genome. Therefore, these LD estimates should be treated with caution. This swift decay suggests a historically large effective population size and high recombination rate, reflecting substantial genetic diversity within the population (Ellegren and Galtier, 2016). Moreover, LD values are population-specific, and influenced by evolutionary factors such as natural selection, mutation, genetic drift, origin and migration, as well as molecular forces including historical recombination events, and breeding history such as historical effective population sizes, intensity and direction of artificial selection, population admixture, and mating patterns (Du et al., 2007). Our findings confirm the low LD in *M. edulis* populations, consistent with previous studies on bivalves (Jones et al., 2013b; Vera et al., 2022; Jourdan et al., 2023). However, the sparse SNP distribution and generally low LD values (*r* ^2^ < 0.1) suggest that increasing marker density is essential to improve prediction accuracy and QTL detection.

### 4.3 Population structure

F_ST_ is widely applied to evaluate genetic differentiation between/among populations (Hu et al., 2022). The low F_ST_ values (F_ST_ < 0.03) observed in our study suggest minimal genetic differentiation among mussel populations, indicating a lack of significant genetic structure. This phenomenon may be attributed to similar selection pressure and limited gene flow among the mussel populations, irrespective of geographic location. Similar to our findings, wild populations of *M. edulis* sampled from the Atlantic coast of France and from the Northern France to Germany had low Fst value (<0.003) using ancestry-informative SNPs (Simon et al., 2020). In addition, similar findings have been reported in other studies, such as pairwise F_ST_ (<0.02) among wild edible cockle using SNPs information (Vera et al., 2022) and among wild populations of Pacific oyster using allozymes and microsatellites markers (Appleyard and Ward, 2006). The PCA result showed that the WIM breeders formed a substructure with allele frequencies that differed from those of the other populations. This divergence may reduce the accuracy of EBV prediction in WIM populations and may be a cause of masking of the genetic architecture, as observed in a study of resistance to nodavirosis in sea bass (Griot et al., 2021). However, this clustering is inconsistent with the low mean F_ST_ values (<0.03), suggesting limited genetic differentiation and a degree of genetic relatedness among the populations. The two families whose offspring showed greater isolation from others in the WIM population may be due to the peculiar characteristics of the parents, which drive the first axis of the PCA.

### 4.4 Heritability

Our study presents the first report of heritability estimates for resistance to *V. splendidus* experimental infection in *M. edulis* based on genome-wide SNPs. We observed moderate heritability for *V. splendidus* resistance (0.22–0.36), which are higher compared to our previous study using the same population. This increase may be attributed to the inclusion of a third experimental infection in this study, despite the overall lower mortality rate (Ajithkumar et al., 2024b). Disease resistance to pathogens in bivalves seems to be a heritable trait, with moderate to high heritability in oysters, clams, and abalone, ranging from 0.21 to 0.63 (Degremont et al., 2015; Brokordt et al., 2017; Smits et al., 2020). Studies on oysters have shown varying levels of heritability (h^2^: 0.09–0.54) against different *Vibrio spp*. pathogens at different life stages (Azema et al., 2017; Nordio et al., 2021; Zhai et al., 2021; Dietrich et al., 2022). When comparing heritability estimates across different methods, both GBLUP and ssGBLUP consistently showed higher heritability estimates than pedigree-based methods. This difference is likely due to the genomic relationship matrix constructed based on genome-wide SNPs information can capture both within and between-family genetic variance, whereas traditional pedigree-based selection only captures genetic variance between families (Boudry et al., 2021). The higher heritability was observed with GBLUP, which may be due to the selective genotyping approach (348 dead and 348 alive individuals genotyped), potentially biasing heritability estimates and limiting the capture of family genetic variation. However, the ssGBLUP approach, which includes both genotyped and non-genotyped individuals, provides a more precise heritability estimate by capturing family variation from both genotyped and phenotyped individuals. To date, numerous studies across aquaculture species have similarly demonstrated that GBLUP methods provide higher estimated heritability and greater accuracy compared to PBLUP (Tsai et al., 2015; Gutierrez et al., 2018). These results underscore the presence of genetic variation for resistance to *V. splendidus* in our mussel populations, and highlight significant opportunities for enhancing disease resistance through selective breeding programs, whether using pedigree-based or genomic selection strategies.

### 4.5 Genome-wide association study

QTL detection in our populations posed challenges due to limited number of markers and individuals. Given the data in the current study do suggest a polygenic nature of resistance to *V. splendidus*, exploiting all markers to calculate genomic breeding values for resistance may be the most effective approach. Our association analyses suggest that resistance against *V. splendidus* exhibits a polygenic architecture without major QTLs. Similar findings have been reported for bacterial disease resistance in various aquaculture species including, Atlantic salmon *Salmo salar* (Correa et al., 2015), Coho salmon *Oncorhynchus kisutch* (Barría et al., 2018), Gilthead Sea Bream (Palaiokostas et al., 2016), European seabass (Oikonomou et al., 2022), and Pacific Oyster (Yang et al., 2022). For instance, a study on catfish identified four QTLs associated with columnaris resistance using a high-density SNP array (Geng et al., 2015), highlighting the importance of high-density SNP array for GWAS studies. Furthermore, multiple studies on rainbow trout concluded that a panel of approximately 5,000 evenly distributed SNPs across the genome was effective in identifying multiple QTLs for resistance to bacterial cold water disease due to the strong long-range LD in breeding populations (Vallejo et al., 2017; Vallejo et al., 2018; Vallejo et al., 2022). The SNP array used in this study had a limited number and quality of SNPs, and the mussel genome showed a very low level of LD. Our study used 2,204 SNPs, which may not provide sufficient coverage given the rapid LD decay and unevenly distributed SNPs across the genome, potentially leading to the omission of important QTLs. Future studies would benefit from incorporating high-quality SNP markers (>30K SNPs) and a larger number of phenotyped individuals (>1,000), which could significantly enhance the power and precision of QTL detection for *V. splendidus* resistance in blue mussels (Jones et al., 2013b; Barría et al., 2018). Additionally, developing an F2 generation by crossing parental lines with clearly contrasting phenotypic traits (resistant and susceptible) would provide an ideal basis for QTL mapping (Gu et al., 2011). In F2 populations, genotype segregation combined with divergent phenotypic differences provides strong conditions for QTL identification (Geng et al., 2015). Family-based mapping in these populations further strengthens QTL detection power by minimizing recombination between QTLs and linked markers, thereby improving the accuracy of marker-trait associations (Geng et al., 2015; Gonen et al., 2015). Moreover, family-based association mapping is particularly effective for detecting rare alleles associated with QTLs, which may be challenging to identify in other population structures (Geng et al., 2015).

### 4.6 Prediction accuracy

The accuracy of genomic selection is affected by several factors, including the relationship between training and validation animals, sample size in the reference population, marker density, effective population size, LD structure, underlying trait architecture and heritability of the trait (Yáñez et al., 2023). Therefore, the lower prediction accuracies observed here may reflect the marker density or underlying genetic architecture of the trait. The choice of genomic selection model for breeding programs requires a prior understanding of the genetic architecture of the selected trait(s). In the current study on *M. edulis* populations, the genetic contribution to the observed variation in resistance to *V. splendidus* was largely polygenic in nature. For the improvement of polygenic traits, GBLUP is the most reliable model and typically provides the highest prediction accuracy for highly polygenic traits, while the Bayesian models are preferable for traits controlled by few large effect loci in genomic selection (Legarra et al., 2015; Yáñez et al., 2023).

In our study, genomic selection improves accuracy of up to 19% compared to pedigree selection. A key consideration for the commercial implementation of genomic selection in shellfish aquaculture is the high cost of genotyping. Reference population size and marker density are two key factors for effectively reducing the cost of genomic selection (Song et al., 2022). One strategy to enhance the economic viability of genomic selection is to use a low-density SNP panel, which can reduce costs while achieving prediction accuracy similar to that of medium-density panels (Kriaridou et al., 2020). The prediction accuracies for genomic models in our study ranged from 0.32 to 0.48 for resistance to *V. splendidus* (with SNP densities ranging from 500 to ∼3,400), whereas the accuracy of PBLUP was 0.36. This result is slightly lower than the ranges reported for disease-related traits in other bivalve species. For instance, genomic selection prediction accuracies from GBLUP models for resistance to Ostreid herpesvirus (OsHV-1-lvar) ranged from 0.68 to 0.76 in the Pacific oyster (Gutierrez et al., 2020). Prediction accuracies for growth-related traits using the GBLUP model in other bivalves are relatively similar, e.g., 0.52–0.73 in the Pacific oyster (Gutierrez et al., 2018; Jourdan et al., 2023), 0.67–0.79 in the Portuguese oyster (Vu et al., 2021), 0.25–0.48 in the Zhikong scallop (Wang et al., 2016) and >0.83 in European flat oyster (Penaloza et al., 2022). Other reports on genomic prediction accuracies for disease-related traits in finfish aquaculture species show the prediction accuracies as low as 0.21, reviewed in Houston et al. (2020). However, this result highlight that genomic selection is a useful approach to increase resistance to *V. splendidus* in our blue mussel populations.

Overall, our results showed that genomic methods predict better accuracy (25%–33%) for resistance to *V. splendidus* using ∼2000 SNPs in a family-based design compared to pedigree-based estimation. This indicates that substantial improvements in the rate of genetic gain can be achieved through genomics-based selection techniques. It also increases the feasibility of using a low-density SNP panel for genomic selection in mussel breeding for *Vibrio* resistance, as low-density genotyping is significantly more cost-effective than high-density SNP arrays. Furthermore, studies on disease resistance in the Pacific oyster, growth traits in the European flat oyster, and heat tolerance in the Pacific abalone have shown that low-density SNP panels of around 1,000–2000 SNPs can achieve EBV accuracies similar to those obtained with medium-density arrays (Gutierrez et al., 2020; Kriaridou et al., 2020; Liu et al., 2022; Penaloza et al., 2022). Similar findings in multiple aquatic species have shown that low-density panels can achieve higher accuracies than the pedigree-based approach, making them a viable alternative for identifying candidates with the highest genetic merit for complex traits such as growth and disease resistance (Kriaridou et al., 2020).

Although the mussel genome is 1.4 Gb in size, our study suggests that a relatively low number of genetic markers can still achieve high prediction accuracy, with a rapid LD decay observed across all populations. In this study, we highlight the possibility of reducing the genotyping costs associated with genomic prediction approaches, caution should be exercised regarding the smallest marker density. Our study found that using only 500 SNPs in the GBLUP model resulted in an estimated decrease in the accuracy of genomic breeding values (GEBVs) for resistance to *V. splendidus* by 11% compared to PBLUP. It is important to note that when both pedigree information and genotypes are available, using ssGBLUP is preferable, as it demonstrates superior accuracy compared to PBLUP. Furthermore, our findings emphasize that annotated SNPs on the *M. edulis* genome provided more information about the studied population and led to higher prediction accuracy than using all SNPs in either the GBLUP or ssGBLUP model. This difference could be due to even distribution of phenotypes among genotyped individuals (50% mortality), or unannotated markers may introduce noise, thereby affecting the accuracy of GEBV estimation. Further investigations using more SNPs (>10K SNPs), and larger reference population (>1,000 individuals) hold potential for genomic selection to further increase the prediction accuracy for host resistance to *V. splendidus* in farmed mussel populations.

## 5 Conclusion

Our study estimated moderate heritability for resistance to *V. splendidus* in blue mussel populations using both pedigree and genomic data from a challenge experiment. GWAS analysis indicates that resistance to *V. splendidus* appears to be polygenic, suggesting that genomic selection is likely to be more effective than marker-assisted selection. We found that genomic selection can improve accuracy by up to 19% compared to pedigree-based selection. Additionally, our results highlight the potential for reducing the number of markers, which could make genomic selection more cost-effective. However, better genomic tools are essential, as the existing SNP arrays lack number of high-quality SNPs, leading to inadequate genome coverage and density. Increasing the number of genotyped individuals is crucial for boosting GWAS power and prediction accuracy. Overall, selective breeding emerges as a promising approach for improving resistance to *V. splendidus* in blue mussels, with genomic selection offering substantial potential for increasing genetic gains.

## Data Availability

The data presented in the study are deposited in the Sextant (Ifremer) repository as independent phenotype and genotype datasets accessible at https://doi.org/10.12770/e4707865-a8e3-4c9f-af99-e37bcd2f507d.

## References

[B1] Aguilar I. Misztal I. Johnson D. L. Legarra A. Tsuruta S. Lawlor T. J. (2010). Hot topic: a unified approach to utilize phenotypic, full pedigree, and genomic information for genetic evaluation of Holstein final score. J. Dairy Sci. 93 (2), 743–752. 10.3168/jds.2009-2730 20105546

[B2] Ajasa A. A. Boison S. A. Gjoen H. M. Lillehammer M. (2024). Accuracy of genomic prediction using multiple Atlantic salmon populations. Genet. Sel. Evol. 56 (1), 38. 10.1186/s12711-024-00907-5 38750427 PMC11094890

[B3] Ajithkumar M. Dégremont L. Garcia C. Ledu C. Benabdelmouna A. (2025). Response to selection for cytogenetic status and their relationship with mortality in Mytilus edulis and Mytilus galloprovincialis in France. Aquaculture 597, 741912. 10.1016/j.aquaculture.2024.741912

[B4] Ajithkumar M. Lillehammer M. Travers M.-A. Maurouard E. Aslam M. L. Dégremont L. (2024b). Genetic parameters for resistance to field mortality outbreaks and resistance to a pathogenic strain of Vibrio splendidus in *Mytilus edulis*, *Mytilus galloprovincialis* and natural hybrid. Aquaculture 590 (0044-8486), 741034. 10.1016/j.aquaculture.2024.741034

[B5] Alcapán A. C. Nespolo R. F. Toro J. E. (2007). Heritability of body size in the Chilean blue mussel (*Mytilus chilensis*Hupé 1854): effects of environment and ageing: heritability of body size: effects of environment and aging. Aquac. Res. 38 (3), 313–320. 10.1111/j.1365-2109.2007.01678.x

[B6] Allam B. Raftos D. (2015). Immune responses to infectious diseases in bivalves. J. Invertebr. Pathol. 131, 121–136. 10.1016/j.jip.2015.05.005 26003824

[B7] Appleyard S. A. Ward R. D. (2006). Genetic diversity and effective population size in mass selection lines of Pacific oyster (*Crassostrea gigas*). Aquaculture 254 (1-4), 148–159. 10.1016/j.aquaculture.2005.10.017

[B8] Azema P. Lamy J. B. Boudry P. Renault T. Travers M. A. Degremont L. (2017). Genetic parameters of resistance to Vibrio aestuarianus, and OsHV-1 infections in the Pacific oyster, *Crassostrea gigas*, at three different life stages. Genet. Sel. Evol. 49, 23. 10.1186/s12711-017-0297-2 28201985 PMC5311879

[B9] Bai Z. Y. Li Q. Q. Han X. K. Li J. L. (2017). Estimates of genetic parameters and genotype by environment interactions for shell nacre color and growth traits in the purple freshwater pearl mussel Hyriopsis cumingii. Aquacult Int. 25 (6), 2079–2090. 10.1007/s10499-017-0170-x

[B10] Barría A. Christensen K. A. Yoshida G. M. Correa K. Jedlicki A. Lhorente J. P. (2018). Genomic predictions and genome-wide association study of resistance against piscirickettsia salmonis in Coho salmon (*Oncorhynchus kisutch*) using ddRAD sequencing. G3-Genes Genom Genet. 8 (4), 1183–1194. 10.1534/g3.118.200053 PMC5873909 29440129

[B11] Bechemin C. Soletchnik P. Polsenaere P. Le Moine O. Pernet F. Protat M. (2015). Episodes de mortalité massive de moules bleues observés en 2014 dans les Pertuis charentais. Bull. Epidémiologie, Santé animale alimentaion (67), 6–9.

[B12] Begum F. Ghosh D. Tseng G. C. Feingold E. (2012). Comprehensive literature review and statistical considerations for GWAS meta-analysis. Nucleic Acids Res. 40 (9), 3777–3784. 10.1093/nar/gkr1255 22241776 PMC3351172

[B13] Ben Cheikh Y. Travers M. A. Le Foll F. (2017). Infection dynamics of a V. splendidus strain pathogenic to *Mytilus edulis*: *in vivo* and *in vitro* interactions with hemocytes. Fish. Shellfish Immun. 70, 515–523. 10.1016/j.fsi.2017.09.047 28935598

[B14] Ben Cheikh Y. Travers M. A. Morga B. Godfrin Y. Le Foll F. (2016). First evidence for a Vibrio strain pathogenic to *Mytilus edulis* altering hemocyte immune capacities. Fish. Shellfish Immun. 53, 91. 10.1016/j.fsi.2016.03.140 26719026

[B15] Bódis E. Tóth B. Sousa R. (2014). Massive mortality of invasive bivalves as a potential resource subsidy for the adjacent terrestrial food web. Hydrobiologia 735 (1), 253–262. 10.1007/s10750-013-1445-5

[B16] Boudry P. Allal F. Aslam M. L. Bargelloni L. Bean T. P. Brard-Fudulea S. (2021). Current status and potential of genomic selection to improve selective breeding in the main aquaculture species of International Council for the Exploration of the Sea (ICES) member countries. Aquac. Rep. 20, 100700. 10.1016/j.aqrep.2021.100700

[B17] Brokordt K. González R. Farías W. Winkler F. E. Lohrmann K. B. (2017). First insight into the heritable variation of the resistance to infection with the bacteria causing the withering syndrome disease in *Haliotis rufescens* abalone. J. Invertebr. Pathol. 150, 15–20. 10.1016/j.jip.2017.08.014 28870439

[B18] Burdon D. Callaway R. Elliott M. Smith T. Wither A. (2014). Mass mortalities in bivalve populations: a review of the edible cockle *Cerastoderma edule* (L.). Estuar. Coast Shelf S 150, 271–280. 10.1016/j.ecss.2014.04.011

[B19] Camara M. D. Symonds J. E. (2014). Genetic improvement of New Zealand aquaculture species: programmes, progress and prospects. New Zeal J. Mar. Fresh 48 (3), 466–491. 10.1080/00288330.2014.932291

[B20] Capelle J. J. Garcia A. B. Kamermans P. Engelsma M. Y. Jansen H. M. (2021). Observations on recent mass mortality events of marine mussels in the Oosterschelde, The Netherlands. Aquacult Int. 29 (4), 1737–1751. 10.1007/s10499-021-00713-6

[B21] Chang C. C. Chow C. C. Tellier L. C. A. M. Vattikuti S. Purcell S. M. Lee J. J. (2015). Second-generation PLINK: rising to the challenge of larger and richer datasets. Gigascience 4, 7. 10.1186/s13742-015-0047-8 25722852 PMC4342193

[B22] Charles M. Trancart S. Oden E. Houssin M. (2020). Experimental infection of *Mytilus edulis* by two Vibrio splendidus-related strains: determination of pathogenicity level of strains and influence of the origin and annual cycle of mussels on their sensitivity. J. Fish. Dis. 43 (1), 9–21. 10.1111/jfd.13094 31659783

[B23] Correa K. Bangera R. Figueroa R. Lhorente J. P. Yáñez J. M. (2017). The use of genomic information increases the accuracy of breeding value predictions for sea louse (Caligus rogercresseyi) resistance in Atlantic salmon (*Salmo salar*). Genet. Sel. Evol. 49, 15. 10.1186/s12711-017-0291-8 28143593 PMC5282780

[B24] Correa K. Lhorente J. P. López M. E. Bassini L. Naswa S. Deeb N. (2015). Genome-wide association analysis reveals loci associated with resistance against Piscirickettsia salmonis in two Atlantic salmon (*Salmo salar* L.) chromosomes. Bmc Genomics 16, 854. 10.1186/s12864-015-2038-7 26499328 PMC4619534

[B25] D'Ambrosio J. Phocas F. Haffray P. Bestin A. Brard-Fudulea S. Poncet C. (2019). Genome-wide estimates of genetic diversity, inbreeding and effective size of experimental and commercial rainbow trout lines undergoing selective breeding. Genet. Sel. Evol. 51, 26. 10.1186/s12711-019-0468-4 31170906 PMC6554922

[B26] Davey J. W. Hohenlohe P. A. Etter P. D. Boone J. Q. Catchen J. M. Blaxter M. L. (2011). Genome-wide genetic marker discovery and genotyping using next-generation sequencing. Nat. Rev. Genet. 12 (7), 499–510. 10.1038/nrg3012 21681211

[B27] Degremont L. Ernande B. Bedier E. Boudry P. (2007). Summer mortality of hatchery-produced Pacific oyster spat (*Crassostrea gigas*). I. Estimation of genetic parameters for survival and growth. Aquaculture 262 (1), 41–53. 10.1016/j.aquaculture.2006.10.025

[B28] Degremont L. Garcia C. Allen S. K. (2015). Genetic improvement for disease resistance in oysters: a review. J. Invertebr. Pathol. 131, 226–241. 10.1016/j.jip.2015.05.010 26037230

[B29] Degremont L. Maurouard E. Rabiller M. Glize P. (2019). Response to selection for increasing resistance to the spring mortality outbreaks in *Mytilus edulis* occurring in France since 2014. Aquaculture 511, 734269. 10.1016/j.aquaculture.2019.734269

[B119] Dempster R.E. Lerner I.M. (1950). Heritability of threshold characters. Genetics 35 (2), 212-236. 10.1093/genetics/35.2.212 17247344 PMC1209482

[B30] Díaz-Puente B. Guiñez R. Pita A. Miñambres M. Presa P. (2020). Genotype by environment interaction for shell length in *Mytilus galloprovincialis* . J. Exp. Mar. Biol. Ecol. 522, 151252. 10.1016/j.jembe.2019.151252

[B31] Dietrich J. P. Hicks M. B. R. Hard J. J. Nichols K. M. Langdon C. J. Divilov K. (2022). Heritability estimates of disease resistance to Vibrio coralliiyticus in Pacific oyster (*Crassostrea gigas*) larvae from a selective broodstock program. Aquaculture 560, 738492. 10.1016/j.aquaculture.2022.738492

[B32] Du F. X. Clutter A. C. Lohuis M. M. (2007). Characterizing linkage disequilibrium in pig populations. Int. J. Biol. Sci. 3 (3), 166–178. 10.7150/ijbs.3.166 17384735 PMC1802018

[B33] Ellegren H. Galtier N. (2016). Determinants of genetic diversity. Nat. Rev. Genet. 17 (7), 422–433. 10.1038/nrg.2016.58 27265362

[B34] Ericson J. A. Venter L. Copedo J. S. Nguyen V. T. Alfaro A. C. Ragg N. L. C. (2023). Chronic heat stress as a predisposing factor in summer mortality of mussels, *Perna canaliculus* . Aquaculture 564, 738986. 10.1016/j.aquaculture.2022.738986

[B35] FAO (2024). The state of world fisheries and aquaculture 2024 – blue transformation in action. Rome.

[B36] Gebreyesus G. Buitenhuis A. J. Poulsen N. A. Visker M. H. P. W. Zhang Q. van Valenberg H. J. F. (2019). Combining multi-population datasets for joint genome-wide association and meta-analyses: the case of bovine milk fat composition traits. J. Dairy Sci. 102 (12), 11124–11141. 10.3168/jds.2019-16676 31563305

[B37] Geng X. Sha J. Liu S. K. Bao L. S. Zhang J. R. Wang R. J. (2015). A genome-wide association study in catfish reveals the presence of functional hubs of related genes within QTLs for columnaris disease resistance. Bmc Genomics 16, 196. 10.1186/s12864-015-1409-4 25888203 PMC4372039

[B38] Gerdol M. Moreira R. Cruz F. Gomez-Garrido J. Vlasova A. Rosani U. (2020). Massive gene presence-absence variation shapes an open pan-genome in the Mediterranean mussel. Genome Biol. 21 (1), 275. 10.1186/s13059-020-02180-3 33168033 PMC7653742

[B39] Gilmour A. R. Thompson R. Cullis B. R. (1995). Average information REML: an efficient algorithm for variance parameter estimation in linear mixed models. Biometrics 51 (4), 1440–1450. 10.2307/2533274

[B40] Gjedrem T. Rye M. (2018). Selection response in fish and shellfish: a review. Rev. Aquacult 10 (1), 168–179. 10.1111/raq.12154

[B41] Goddard M. E. Hayes B. J. (2009). Mapping genes for complex traits in domestic animals and their use in breeding programmes. Nat. Rev. Genet. 10 (6), 381–391. 10.1038/nrg2575 19448663

[B42] Gonen S. Baranski M. Thorland I. Norris A. Grove H. Arnesen P. (2015). Mapping and validation of a major QTL affecting resistance to pancreas disease (salmonid alphavirus) in Atlantic salmon (*Salmo salar*). Heredity 115 (5), 405–414. 10.1038/hdy.2015.37 25990876 PMC4611234

[B43] Griot R. Allal F. Brard-Fudulea S. Morvezen R. Haffray P. Phocas F. (2020). APIS: an auto-adaptive parentage inference software that tolerates missing parents. Mol. Ecol. Resour. 20 (2), 579–590. 10.1111/1755-0998.13103 31609085

[B44] Griot R. Allal F. Phocas F. Brard-Fudulea S. Morvezen R. Bestin A. (2021). Genome-wide association studies for resistance to viral nervous necrosis in three populations of European sea bass (*Dicentrarchus labrax*) using a novel 57k SNP array DlabChip. Aquaculture 530, 735930. 10.1016/j.aquaculture.2020.735930

[B45] Gu X. Feng C. Ma L. Song C. Wang Y. Da Y. (2011). Genome-wide association study of body weight in chicken F2 resource population. Plos One 6 (7), e21872. 10.1371/journal.pone.0021872 21779344 PMC3136483

[B46] Guiñez R. Toro J. E. Krapivka S. Alcapán A. C. Oyarzún P. A. (2017). Heritabilities and genetic correlation of shell thickness and shell length growth in a mussel, Mytilus chilensis (Bivalvia:Mytilidae). Aquac. Res. 48 (4), 1450–1457. 10.1111/are.12981

[B47] Guo X. M. Puritz J. B. Wang Z. W. Proestou D. Allen S. Small J. (2023). Development and evaluation of high-density SNP arrays for the eastern oyster *Crassostrea virginica* . Mar. Biotechnol. 25 (1), 174–191. 10.1007/s10126-022-10191-3 36622459

[B48] Gutierrez A. P. Matika O. Bean T. P. Houston R. D. (2018). Genomic selection for growth traits in pacific oyster (*Crassostrea gigas*): potential of low-density marker panels for breeding value prediction. Front. Genet. 9, 391. 10.3389/fgene.2018.00391 30283494 PMC6156352

[B49] Gutierrez A. P. Symonds J. King N. Steiner K. Bean T. P. Houston R. D. (2020). Potential of genomic selection for improvement of resistance to ostreid herpesvirus in Pacific oyster (*Crassostrea gigas*). Anim. Genet. 51 (2), 249–257. 10.1111/age.12909 31999002

[B50] Gutierrez A. P. Turner F. Gharbi K. Talbot R. Lowe N. R. Peñaloza C. (2017). Development of a medium density combined-species SNP array for pacific and European oysters (*Crassostrea gigas* and *Ostrea edulis*). G3-Genes Genom Genet. 7 (7), 2209–2218. 10.1534/g3.117.041780 PMC5499128 28533337

[B116] Hedgecock D. Shin G. Gracey A. Y. Van Den Berg D. Samanta M. P. (2015). Second-Generation Linkage Maps for the Pacific Oyster Crassostrea gigas Reveal Errors in Assembly of Genome Scaffolds. G3-Genes Genomes Genetics 5, 2007–2019. 10.1534/g3.115.019570 26248981 PMC4592983

[B51] Hollenbeck C. M. Johnston I. A. (2018). Genomic tools and selective breeding in molluscs. Front. Genet. 9, 253. 10.3389/fgene.2018.00253 30073016 PMC6058216

[B52] Houston R. D. Bean T. P. Macqueen D. J. Gundappa M. K. Jin Y. H. Jenkins T. L. (2020). Harnessing genomics to fast-track genetic improvement in aquaculture. Nat. Rev. Genet. 21 (7), 389–409. 10.1038/s41576-020-0227-y 32300217

[B53] Hu Y. M. Li Q. Xu C. X. Liu S. K. Kong L. F. Yu H. (2022). Genetic variability of mass-selected and wild populations of Iwagaki oyster (Crassostrea nippona) revealed by microsatellites and mitochondrial COI sequences. Aquaculture 561, 738737. 10.1016/j.aquaculture.2022.738737

[B54] Jiao W. Q. Fu X. T. Dou J. Z. Li H. D. Su H. L. Mao J. X. (2014). High-resolution linkage and quantitative trait locus mapping aided by genome survey sequencing: building up an integrative genomic framework for a bivalve mollusc. DNA Res. 21 (1), 85–101. 10.1093/dnares/dst043 24107803 PMC3925396

[B55] Jiao Z. X. Tian Y. Hu B. Y. Li Q. Liu S. K. (2021). Genome structural variation landscape and its selection signatures in the fast-growing strains of the pacific oyster, *Crassostrea gigas* . Mar. Biotechnol. 23 (5), 736–748. 10.1007/s10126-021-10060-5 34498173

[B56] Jin W. Bai Z. Y. Fu L. L. Zhang G. F. Li J. L. (2012). Genetic analysis of early growth traits of the triangle shell mussel, Hyriopsis Cumingii, as an insight for potential genetic improvement to pearl quality and yield. Aquacult Int. 20 (5), 927–933. 10.1007/s10499-012-9518-4

[B57] Jones D. B. Jerry D. R. Forêt S. Konovalov D. A. Zenger K. R. (2013a). Genome-wide SNP validation and mantle tissue transcriptome analysis in the silver-lipped pearl oyster, pinctada maxima. Mar. Biotechnol. 15 (6), 647–658. 10.1007/s10126-013-9514-3 23715808

[B58] Jones D. B. Jerry D. R. Khatkar M. S. Raadsma H. W. Zenger K. R. (2013b). A high-density SNP genetic linkage map for the silver-lipped pearl oyster, Pinctada maxima: a valuable resource for gene localisation and marker-assisted selection. Bmc Genomics 14, 810. 10.1186/1471-2164-14-810 24252414 PMC4046678

[B59] Jourdan A. Morvezen R. Enez F. Haffray P. Lange A. Vetois E. (2023). Potential of genomic selection for growth, meat content and colour traits in mixed-family breeding designs for the Pacific oyster *Crassostrea gigas* . Aquaculture 576, 739878. 10.1016/j.aquaculture.2023.739878

[B60] Kriaridou C. Tsairidou S. Houston R. D. Robledo D. (2020). Genomic prediction using low density marker panels in aquaculture: performance across species, traits, and genotyping platforms. Front. Genet. 11, 124. 10.3389/fgene.2020.00124 32174974 PMC7056899

[B61] Kristensen P. S. Jahoor A. Andersen J. R. Cericola F. Orabi J. Janss L. L. (2018). Genome-wide association studies and comparison of models and cross-validation strategies for genomic prediction of quality traits in advanced winter wheat breeding lines. Front. Plant Sci. 9, 69. 10.3389/fpls.2018.00069 29456546 PMC5801407

[B62] Legarra A. Croiseau P. Sanchez M. P. Teyssèdre S. Sallé G. Allais S. (2015). A comparison of methods for whole-genome QTL mapping using dense markers in four livestock species. Genet. Sel. Evol. 47, 6. 10.1186/s12711-015-0087-7 25885597 PMC4324410

[B63] Liu J. Y. Peng W. Z. Yu F. Shen Y. W. Yu W. C. Lu Y. S. (2022). Genomic selection applications can improve the environmental performance of aquatics: a case study on the heat tolerance of abalone. Evol. Appl. 15 (6), 992–1001. 10.1111/eva.13388 35782008 PMC9234619

[B64] Lund M. S. de Roos A. P. W. de Vries A. G. Druet T. Ducrocq V. Fritz S. (2011). A common reference population from four European Holstein populations increases reliability of genomic predictions. Genet. Sel. Evol. 43 (1), 43. 10.1186/1297-9686-43-43 22152008 PMC3292506

[B65] Lupo C. Bougeard S. Le Bihan V. Blin J. L. Allain G. Azema P. (2021). Mortality of marine mussels *Mytilus edulis* and *M. galloprovincialis*: systematic literature review of risk factors and recommendations for future research. Rev. Aquacult 13 (1), 504–536. 10.1111/raq.12484

[B66] Mallet A. L. Freeman K. R. Dickie L. M. (1986). The genetics of production characters in the blue mussel *Mytilus edulis*. I. A preliminary analysis. Aquaculture 57 (1-4), 133–140. 10.1016/0044-8486(86)90190-0

[B67] McCarty A. J. Allen S. K. Plough L. V. (2022). Genome-wide analysis of acute low salinity tolerance in the eastern oyster *Crassostrea virginica* and potential of genomic selection for trait improvement. G3-Genes Genom Genet. 12 (1), jkab368. 10.1093/g3journal/jkab368 PMC8727987 34849774

[B68] Misztal I. Tsuruta S. Lourenco D. A. L. Aguilar I. Legarra A. Vitezica Z. (2014). BLUPF90 and related programs (BGF90).

[B69] Nascimento-Schulze J. C. Bean T. P. Penaloza C. Paris J. R. Whiting J. R. Simon A. (2023). SNP discovery and genetic structure in blue mussel species using low coverage sequencing and a medium density 60 K SNP-array. Evol. Appl. 16 (5), 1044–1060. 10.1111/eva.13552 37216031 PMC10197230

[B70] Nguyen T. T. T. Hayes B. J. Ingram B. A. (2014). Genetic parameters and response to selection in blue mussel (*Mytilus galloprovincialis*) using a SNP-based pedigree. Aquaculture 420, 295–301. 10.1016/j.aquaculture.2013.11.021

[B71] Nie H. T. Yan X. W. Huo Z. M. Jiang L. W. Chen P. Liu H. (2017). Construction of a high-density genetic map and quantitative trait locus mapping in the manila clam ruditapes philippinarum. Sci. Rep-Uk 7, 229. 10.1038/s41598-017-00246-0 PMC5427961 28331182

[B72] Nordio D. Khtikian N. Andrews S. Bertotto D. Leask K. Green T. (2021). Adaption potential of *Crassostrea gigas* to ocean acidification and disease caused by Vibrio harveyi. Ices J. Mar. Sci. 78 (1), 360–367. 10.1093/icesjms/fsaa080

[B73] Norman R. A. Crumlish M. Stetkiewicz S. (2019). The importance of fisheries and aquaculture production for nutrition and food security. Rev. Sci. Tech. Oie 38 (2), 395–407. 10.20506/rst.38.2.2994 31866686

[B74] Normand J. Benabdelmouna A. Louis W. Grizon J. (2022). MYTILOBS Campagne 2020-2021. Réseau d'observation des moules d'élevage sur la côte Atlantique et dans la Manche. Edition 2022.

[B75] Odegård J. Moen T. Santi N. Korsvoll S. A. Kjoglum S. Meuwissen T. H. E. (2014). Genomic prediction in an admixed population of Atlantic salmon (*Salmo salar*). Front. Genet. 5, 402. 10.3389/fgene.2014.00402 25484890 PMC4240172

[B76] Oden E. Burioli E. A. V. Trancart S. Pitel P. H. Houssin M. (2016). Multilocus sequence analysis of Vibrio splendidus related-strains isolated from blue mussel Mytilus sp during mortality events. Aquaculture 464, 420–427. 10.1016/j.aquaculture.2016.07.024

[B77] Oikonomou S. Samaras A. Tekeoglou M. Loukovitis D. Dimitroglou A. Kottaras L. (2022). Genomic selection and genome-wide association analysis for stress response, disease resistance and body weight in European seabass. Animals 12 (3), 277. 10.3390/ani12030277 35158601 PMC8833606

[B78] Palaiokostas C. Ferraresso S. Franch R. Houston R. D. Bargelloni L. (2016). Genomic prediction of resistance to pasteurellosis in Gilthead Sea Bream (*Sparus aurata*) using 2b-RAD sequencing. G3-Genes Genom Genet. 6 (11), 3693–3700. 10.1534/g3.116.035220 PMC5100868 27652890

[B79] Penaloza C. Barria A. Papadopoulou A. Hooper C. Preston J. Green M. (2022). Genome-wide association and genomic prediction of growth traits in the European flat oyster (*Ostrea edulis*). Front. Genet. 13, 926638. 10.3389/fgene.2022.926638 35983410 PMC9380691

[B80] Pino-Querido A. Alvarez-Castro J. M. Guerra-Varela J. Toro M. A. Vera M. Pardo B. G. (2015). Heritability estimation for okadaic acid algal toxin accumulation, mantle color and growth traits in Mediterranean mussel (*Mytilus galloprovincialis*). Aquaculture 440, 32–39. 10.1016/j.aquaculture.2015.01.032

[B81] Polsenaere P. Soletchnik P. Le Moine O. Gohin F. Robert S. Pepin J. F. (2017). Potential environmental drivers of a regional blue mussel mass mortality event (winter of 2014, Breton Sound, France). J. Sea Res. 123, 39–50. 10.1016/j.seares.2017.03.005

[B82] Prou J. Goulletquer P. (2002). The French mussel industry: present status and perspectives. Bull. Aquac. Assoc. Can. 102 (3), 17–23.

[B83] Qi H. G. Song K. Li C. Y. Wang W. Li B. S. Li L. (2017). Construction and evaluation of a high-density SNP array for the Pacific oyster (*Crassostrea gigas*). Plos One 12 (3), e0174007. 10.1371/journal.pone.0174007 28328985 PMC5362100

[B84] Regan T. Bean T. P. Ellis T. Davie A. Carboni S. Migaud H. (2021). Genetic improvement technologies to support the sustainable growth of UK aquaculture. Rev. Aquacult 13 (4), 1958–1985. 10.1111/raq.12553

[B85] Ren P. Peng W. Z. You W. W. Huang Z. K. Guo Q. Chen N. (2016). Genetic mapping and quantitative trait loci analysis of growth-related traits in the small abalone Haliotis diversicolor using restriction-site-associated DNA sequencing. Aquaculture 454, 163–170. 10.1016/j.aquaculture.2015.12.026

[B86] Resende M. F. R. Muñoz P. Resende M. D. V. Garrick D. J. Fernando R. L. Davis J. M. (2012). Accuracy of genomic selection methods in a standard data set of loblolly pine (pinus taeda L.). Genetics 190 (4), 1503–1510. 10.1534/genetics.111.137026 22271763 PMC3316659

[B87] Rey-Campos M. Moreira R. Gerdol M. Pallavicini A. Novoa B. Figueras A. (2019). Immune tolerance in *Mytilus galloprovincialis* hemocytes after repeated contact with Vibrio splendidus. Front. Immunol. 10, 1894. 10.3389/fimmu.2019.01894 31447861 PMC6697025

[B88] Robledo D. Matika O. Hamilton A. Houston R. D. (2018). Genome-wide association and genomic selection for resistance to amoebic gill disease in atlantic salmon. G3-Genes Genom Genet. 8 (4), 1195–1203. 10.1534/g3.118.200075 PMC5873910 29420190

[B89] Simon A. Arbiol C. Nielsen E. E. Couteau J. Sussarellu R. Burgeot T. (2020). Replicated anthropogenic hybridisations reveal parallel patterns of admixture in marine mussels. Evol. Appl. 13 (3), 575–599. 10.1111/eva.12879 32431737 PMC7045717

[B90] Siol M. Jacquin F. Chabert-Martinello M. Smykal P. Le Paslier M. C. Aubert G. (2017). Patterns of genetic structure and linkage disequilibrium in a large collection of pea germplasm. G3-Genes Genom Genet. 7 (8), 2461–2471. 10.1534/g3.117.043471 PMC5555454 28611254

[B117] Smietanka B. Burzynski A. Hummel H. Wenne R. (2014). Glacial history of the European marine mussels Mytilus, inferred from distribution of mitochondrial DNA lineages. Heredity 113 (3), 250–258. 10.1038/hdy.2014.23 24619178 PMC4815643

[B91] Smits M. Enez F. Ferraresso S. Dalla Rovere G. Vetois E. Auvray J. F. (2020). Potential for genetic improvement of resistance to perkinsus olseni in the manila clam, ruditapes philippinarum, using DNA parentage assignment and mass spawning. Front. Vet. Sci. 7, 579840. 10.3389/fvets.2020.579840 33195590 PMC7649815

[B92] Song H. Guo X. M. Sun L. N. Wang Q. H. Han F. M. Wang H. Y. (2021). The hard clam genome reveals massive expansion and diversification of inhibitors of apoptosis in Bivalvia. Bmc Biol. 19 (1), 15. 10.1186/s12915-020-00943-9 33487168 PMC7831173

[B93] Song H. L. Dong T. Yan X. Y. Wang W. Tian Z. H. Sun A. (2022). Genomic selection and its research progress in aquaculture breeding. Rev. Aquacult 15, 274–291. 10.1111/raq.12716

[B94] Soon T. K. Ransangan J. (2019). Extrinsic factors and marine bivalve mass mortalities: an overview. J. Shellfish Res. 38 (2), 223–232. 10.2983/035.038.0202

[B118] Sun J. Zhang Y. Xu T. Zhang Y. Mu H. Zhang Y. (2017). Adaptation to deep-sea chemosynthetic environments as revealed by mussel genomes. Nat. Ecol. Evol. 1 (5), 0121. 10.1038/s41559-017-0121 28812709

[B95] Suplicy F. M. (2020). A review of the multiple benefits of mussel farming. Rev. Aquacult 12 (1), 204–223. 10.1111/raq.12313

[B96] Tan K. Zhang H. K. Zheng H. P. (2020). Selective breeding of edible bivalves and its implication of global climate change. Rev. Aquacult 12 (4), 2559–2572. 10.1111/raq.12458

[B97] Team P. (2024). RStudio: integrated development environment for R. Posit software. Boston, MA: PBC. URL.

[B98] Toro J. E. Alcapán A. C. Ojeda J. A. Vergara A. M. (2004a). Selection response for growth rate (shell height and live weight) in the Chilean blue mussel (Mytilus chilensis Hupe 1854). J. Shellfish Res. 23 (3), 753–757.

[B99] Toro J. E. Alcapán A. C. Vergara A. M. Ojeda J. A. (2004b). Heritability estimates of larval and spat shell height inthe Chilean blue mussel (Mytilus chilensisHupe 1854)produced under controlled laboratory conditions. Aquac. Res. 35 (1), 56–61. 10.1111/j.1365-2109.2004.00985.x

[B100] Tsai H. Y. Hamilton A. Tinch A. E. Guy D. R. Gharbi K. Stear M. J. (2015). Genome wide association and genomic prediction for growth traits in juvenile farmed Atlantic salmon using a high density SNP array. Bmc Genomics 16, 969. 10.1186/s12864-015-2117-9 26582102 PMC4652364

[B101] Tsuruta S. Misztal I. (2006). THRGIBBS1F90 for estimation of variance components with threshold and linear models. J. Anim. Sci. 84, 15.

[B102] Vallejo R. L. Evenhuis J. P. Cheng H. Fragomeni B. O. Gao G. Liu S. (2022). Genome-wide mapping of quantitative trait loci that can be used in marker-assisted selection for resistance to bacterial cold water disease in two commercial rainbow trout breeding populations. Aquaculture 560, 738574. 10.1016/j.aquaculture.2022.738574

[B103] Vallejo R. L. Liu S. Gao G. Fragomeni B. O. Hernandez A. G. Leeds T. D. (2017). Similar genetic architecture with shared and unique quantitative trait loci for bacterial cold water disease resistance in two rainbow trout breeding populations. Front. Genet. 8, 156. 10.3389/fgene.2017.00156 29109734 PMC5660510

[B104] Vallejo R. L. Silva R. M. O. Evenhuis J. P. Gao G. Liu S. Parsons J. E. (2018). Accurate genomic predictions for BCWD resistance in rainbow trout are achieved using low-density SNP panels: evidence that long-range LD is a major contributing factor. J. Anim. Breed. Genet. 135, 263–274. 10.1111/jbg.12335 29869355

[B105] VanRaden P. M. (2008). Efficient methods to compute genomic predictions. J. Dairy Sci. 91 (11), 4414–4423. 10.3168/jds.2007-0980 18946147

[B106] VanRaden P. M. Van Tassell C. P. Wiggans G. R. Sonstegard T. S. Schnabel R. D. Taylor J. F. (2009). Invited review: reliability of genomic predictions for North American holstein bulls. J. Dairy Sci. 92 (1), 16–24. 10.3168/jds.2008-1514 19109259

[B107] Vendrami D. L. J. Houston R. D. Gharbi K. Telesca L. Gutierrez A. P. Gurney-Smith H. (2019). Detailed insights into pan‐European population structure and inbreeding in wild and hatchery Pacific oysters (*Crassostrea gigas*) revealed by genome‐wide SNP data. Evol. Appl. 12 (3), 519–534. 10.1111/eva.12736 30847007 PMC6383735

[B108] Vera M. Maroso F. Wilmes S. B. Hermida M. Blanco A. Fernández C. (2022). Genomic survey of edible cockle (*Cerastoderma edule*) in the Northeast Atlantic: a baseline for sustainable management of its wild resources. Evol. Appl. 15 (2), 262–285. 10.1111/eva.13340 35233247 PMC8867702

[B109] Vu S. V. Gondro C. Nguyen N. T. H. Gilmour A. R. Tearle R. Knibb W. (2021). Prediction accuracies of genomic selection for nine commercially important traits in the Portuguese oyster (Crassostrea angulata) using DArT-seq technology. Genes 12 (2), 210. 10.3390/genes12020210 33535381 PMC7910873

[B110] Wang J. P. Li L. Zhang G. F. (2016). A high-density SNP genetic linkage map and QTL analysis of growth-related traits in a hybrid family of oysters (*Crassostrea gigas* × Crassostrea angulata) using genotyping-by-sequencing. G3-Genes Genom Genet. 6 (5), 1417–1426. 10.1534/g3.116.026971 PMC4856092 26994291

[B111] Wang Z. Y. Hu H. H. Sun T. Y. Li X. Lv G. L. Bai Z. Y. (2022). Genomic selection for improvement of growth traits in triangle sail mussel (Hyriopsis cumingii). Aquaculture 561, 738692. 10.1016/j.aquaculture.2022.738692

[B112] Yáñez J. M. Barría A. López M. E. Moen T. Garcia B. F. Yoshida G. M. (2023). Genome-wide association and genomic selection in aquaculture. Rev. Aquacult 15 (2), 645–675. 10.1111/raq.12750

[B113] Yang B. Zhai S. Y. Zhang F. Q. Wang H. B. Ren L. T. Li Y. J. (2022). Genome-wide association study toward efficient selection breeding of resistance to Vibrio alginolyticus in Pacific oyster, *Crassostrea gigas* . Aquaculture 548, 737592. 10.1016/j.aquaculture.2021.737592

[B114] Zenger K. R. Khatkar M. S. Jones D. B. Khalilisamani N. Jerry D. R. Raadsma H. W. (2019). Genomic selection in aquaculture: application, limitations and opportunities with special reference to marine shrimp and pearl oysters. Front. Genet. 9, 693. 10.3389/fgene.2018.00693 30728827 PMC6351666

[B115] Zhai S. Y. Yang B. Zhang F. Q. Li Q. Liu S. K. (2021). Estimation of genetic parameters for resistance to Vibrio alginolyticus infection in the Pacific oyster (*Crassostrea gigas*). Aquaculture 538, 736545. 10.1016/j.aquaculture.2021.736545

