# Supplementary material for: Genomic selection for resistance to one pathogenic strain of Vibrio splendidus in blue mussel Mytilus edulis
Source: Front Genet. 2025 Jan 3;15:1487807. doi: 10.3389/fgene.2024.1487807 (PMC11739312; doi:10.3389/fgene.2024.1487807)
Supplement: Supplementary file 2 [file DataSheet1.docx]

Supplementary Table 1: Details of experimental infection (EI_1, EI_2, and EI_3), family mortality per experiment and genotyped individuals

Supplementary Table 2: Number of polyhigh SNPs in each filtration step

| Quality control step | Number of polyhigh SNPs |
| --- | --- |
| Axiom analysis suite | 7476 |
| Plink | 3406 |
| Pedigree assignment | 1471 |
| PCA | 3406 |
| Annotation | 2204 |

Supplementary Table 3: Heritability (diagonal) and genetic correlation (above diagonal) for resistance to *V. splendidus* in *M. edulis* from GBLUP analysis using AIREMLF90

|  | YEU + OLE_PON | WIM |
| --- | --- | --- |
| YEU + OLE_PON | 0.17 (0.08) | 0.99 (0.31) |
| WIM |  | 0.57 (0.16) |

The OLE-PON population did not have enough individuals or families to perform accurate single population univariate model, or bivariate analysis to compare with the other populations. No model was converging in a credible manner, and no results could be interpreted. Since OLE-PON seems to not be very different from YEU (Fst= 0.028), PCA plot (Figure 2) show that they are not separated, and that both populations were sampled at Atlantic coasts, we have decided to combine OLE-PON and YEU for genetic parameter estimation.

Supplementary Table 4: Population specific genotyped and phenotyped individuals with corresponding mean mortality

| Pop/method | Genotyped | Phenotyped | mean mortality |
| --- | --- | --- | --- |
| YEU | 294 | 990 | 45% |
| WIM | 244 | 930 | 54% |
| OLE_PON | 106 | 360 | 37% |

Supplementary Table 5: Summary of the SNP number per each chromosome in blue mussel

| **Chr** | **N** | **Total length (bp)** | **Average SNPs (Mb)** |
| --- | --- | --- | --- |
| 1 | 296 | 124,762,778 | 2.37 |
| 2 | 249 | 107,181,506 | 2.32 |
| 3 | 237 | 105,277,379 | 2.25 |
| 4 | 194 | 97,481,307 | 1.99 |
| 5 | 182 | 96,292,600 | 1.89 |
| 6 | 108 | 90,518,082 | 1.19 |
| 7 | 162 | 89,208,578 | 1.81 |
| 8 | 149 | 89,561,996 | 1.66 |
| 9 | 166 | 86,690,860 | 1.91 |
| 10 | 157 | 83,906,862 | 1.87 |
| 11 | 48 | 83,189,572 | 0.57 |
| 12 | 89 | 78,976,983 | 1.12 |
| 13 | 93 | 70,601,564 | 1.31 |
| 14 | 60 | 68,104,009 | 0.88 |

Chr = Chromosome; N = Number of SNPs


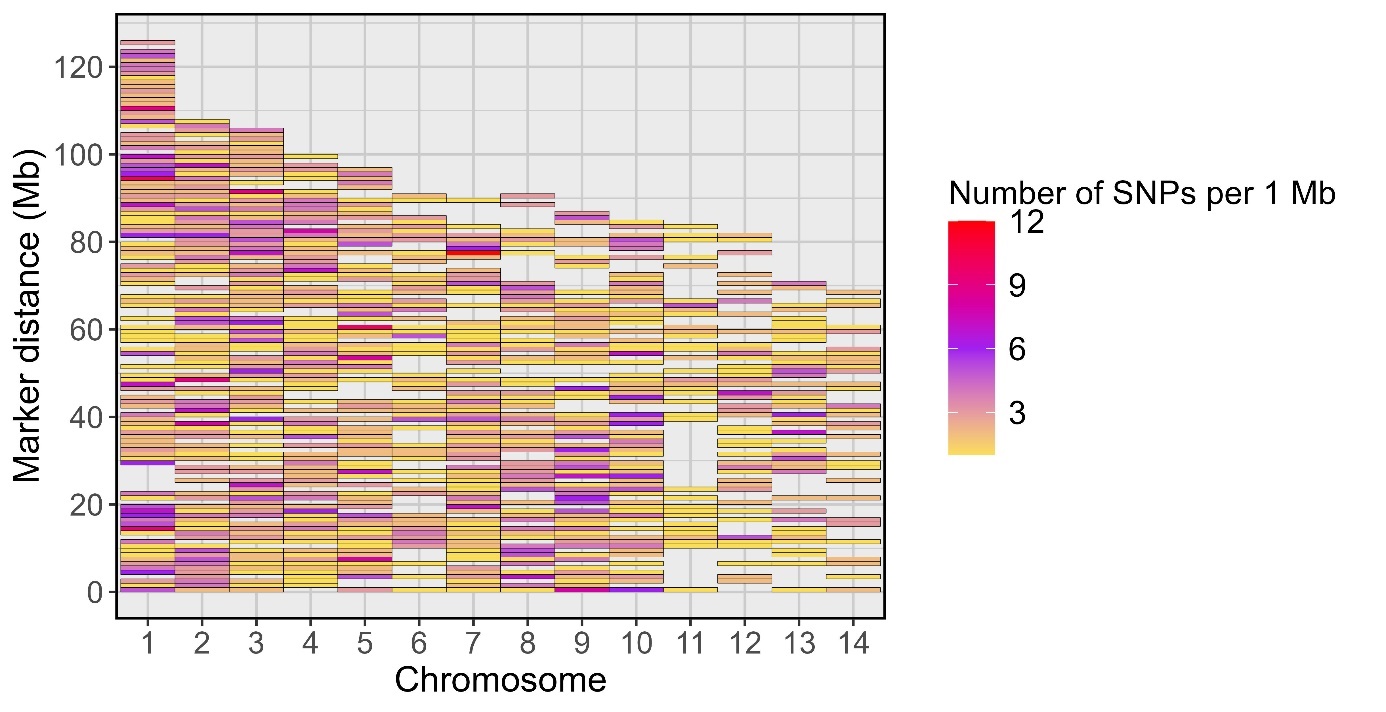


Supplementary Figure 1: Identification of high-quality SNPs and their distribution across the 14 chromosomes of *M. edulis*. The gradient colors from yellow to red denote the increase of SNP density within 1 Mb interval.
